# Colposcopy and Loop Electrosurgical Excision Procedure: A Simulated Exercise

**DOI:** 10.15766/mep_2374-8265.11344

**Published:** 2023-09-08

**Authors:** Cynthia Abraham, Renita Kim, Ceyda Oner, Adjoa Bucknor

**Affiliations:** 1 Attending Physician, Department of Obstetrics, Gynecology, and Reproductive Science, Icahn School of Medicine at Mount Sinai and Mount Sinai Health System

**Keywords:** Colposcopy, LEEP, Clinical/Procedural Skills Training, OB/GYN, Simulation

## Abstract

**Introduction:**

Cervical intraepithelial neoplasia 3 is associated with a high degree of progression to cervical cancer. Its risk is markedly reduced after excisional treatment. Hence, it is critical that providers accurately diagnose and treat this condition. We present a simulation-based module focused on resident mastery of performance of colposcopy and loop electrosurgical excision procedure (LEEP).

**Methods:**

Learners were obstetrics and gynecology residents. Guidelines on performance of colposcopy and LEEP were presented prior to module participation. We used pelvic task trainers, kielbasa sausages, and routine equipment for performance of colposcopy and LEEP. Colposcopy and LEEP sessions each lasted 30 minutes. Learners completed questionnaires before and after regarding comfort level on aspects of colposcopy and LEEP performance and level of agreement with statements on performing procedures independently. Comfort levels and degrees of agreement were based on 5-point Likert scales (1 = *very uncomfortable/strongly disagree,* 3 = *neither comfortable nor uncomfortable/neutral,* 5 = *very comfortable/strongly agree,* respectively).

**Results:**

Modules were held in November 2021 and May 2022. Thirty-four residents participated. Mean comfort scores significantly increased from 3.1 to 4.3 (*p* < .001) before and after the module for all steps. There was an increase in level of agreement with statements on being able to independently perform colposcopy (2.2 to 3.5, *p* < .01) and LEEP (2.9 to 3.6, *p* = .06).

**Discussion:**

Simulation-based modules on performance of colposcopy and LEEP significantly increased resident learner comfort in the performance of these procedures. Comfort in performing these procedures is important in providing comprehensive gynecologic care.

## Educational Objectives

By the end of this module, learners will be able to:

1. Identify indications for colposcopy and loop electrosurgical excision procedure (LEEP).
2. Position and focus the colposcope.
3. Collect cervical biopsies of abnormal areas and perform endocervical curettage.
4. Prepare for LEEP and collect the LEEP specimen.
5. Identify methods for assuring hemostasis after performance of colposcopy and LEEP.

## Introduction

In the United States, cervical cancer incidence and mortality has decreased by 75% since the 1960s. However, this decline has not been uniform and has been influenced by access to care. Worldwide, cervical cancer is the fourth most common cause of cancer in women.^1^ The guiding principle in cervical cancer screening lies in identifying risk of developing cervical intraepithelial neoplasia 3 (CIN3) as opposed to carcinoma. CIN3 has been chosen as an end point due not only to high risk of progression to malignancy but also to its associated risk being markedly reduced after treatment.^2^ Current guidelines take into account cervical cytology on the Papanicolaou smear, high-risk human papilloma virus (HPV) status, and other prognosticators for developing CIN3, such as prior cervical cytology, smoking history, and history of immunosuppression. High-risk HPV is the causative agent of abnormal cervical cytology. A risk level for development of CIN3 is then determined. If the risk of developing CIN3 is equal to (1) 0.55% or less, (2) 4%-24%, (3) 25%-59%, or (4) 60% or greater, one will be advised on the following management options: (1) observation, (2) colposcopy, (3) colposcopy or excisional procedure, and (4) excisional procedure, respectively.

Colposcopy is a procedure in which a device called a colposcope is used to closely examine the cervix with magnification and illumination. It is indicated in the setting of most abnormal cervical cytology results and persistence of presence of high-risk HPV. During colposcopy, acetic acid is applied to the cervix. This dehydrates cells on the cervix and causes those cells with relatively large nuclei to reflect light and appear white. Cells that appear white tend to be those infected with HPV and are termed *aceto-white.* The aceto-white areas of more concern are those in which there is abnormal vasculature traversing through. At the time of colposcopy, biopsies are performed at sites that appear aceto-white and are sent for pathologic evaluation. If there are no lesions that appear white after application of acetic acid, Lugol's solution may then be applied to assure absence of abnormal areas. On application of Lugol's solution, abnormal epithelium appears unstained whereas normal epithelium appears darkly stained. A sampling of the transformation zone of the cervix is also routinely obtained and sent. If pathology indicates presence of CIN3, an excisional procedure is then recommended.^3^

The loop electrosurgical excision procedure (LEEP) is a widely used excisional tool for diagnosis and treatment of CIN3. LEEP is one of the most commonly performed gynecologic procedures. Like colposcopy, it is typically performed in the office. However, complications such as bleeding, infection and perforation have been reported to occur in up to 10% of patients.^4^ During the LEEP, it is imperative not only to excise the lesion but also to remove a sufficient portion of the transformation zone of the cervix as the lesion may extend from the surface of the cervix into the cervical canal. Moreover, it is critical to perform the excision in one motion to prevent fragmentation of the specimen which may necessitate additional procedures. Therefore, resident physicians must achieve mastery of both colposcopy and LEEP. However, the amount of exposure that resident physicians have to these procedures is variable. Additionally, the Accreditation Council for Graduate Medical Education has set no minimum number of colposcopies and/or LEEPs as required for a resident physician to perform in order to graduate from a residency in obstetrics and gynecology. Hence, simulation is an ideal approach to solidifying skills in performance of these procedures.

Literature on resident education in performance of LEEP is limited but provides support for the effectiveness of simulation. A PubMed search using the search words *LEEP* and *simulation* generated seven articles; six presented statistics on efficacy. Hefler and colleagues assessed the efficacy of a module combining didactics and simulation. Knowledge (based on multiple-choice test scores) and LEEP performance (as evaluated by completion of checklists by trained providers) both significantly increased after participation in the module (*p* < .001).^4^ A 2019 study by Takacs and colleagues attested further to the utility of simulation. The authors noted that after five training sessions, accuracy of excision significantly improved (excision depth before training was 7.34 ± 1.60 mm vs. 8.54 ± 1.67 mm after training, *p* = .004) and incidence of fragmentation significantly decreased (2.57 ± 1.26 pieces before training vs. 1.29 ± 0.60 pieces after training, *p* < .001).^5^

Two out of the six articles (including Hefler et al.^4^) evaluated the efficacy of simulation on resident education pertaining to both colposcopy and LEEP. Phoolcharoen and colleagues conducted a study assessing the utility of hands-on training courses in low- and middle-income countries. Five hundred six participants participated across six countries. There was an increase in confidence in performance of colposcopy and LEEP in 71% and 76% of participants, respectively.^6^

Here, we present a simulation-based module focused on providing resident physicians with the skills to perform colposcopy and LEEP. Unlike other published papers, this publication provides specific details to allow for ease of reproduction at other institutions. To date, there is no literature in *MedEdPORTAL* on performance of colposcopy and LEEP. Additionally, this is the first publication that includes a detailed facilitator's guide outlining not only how to construct the models for the session but also how to incorporate presession reading material to reinforce knowledge and skills.

## Methods

### Development

We performed this simulation module in the outpatient ambulatory gynecology center at Mount Sinai Hospital. A detailed facilitator's guide appears in Appendix A.

We required learners to review Appendix B, which outlined indications and steps in performance of colposcopy LEEP, prior to participating in the simulation. We emailed Appendix B to learners prior to the simulation. We obtained all images in Appendix B at the time of the actual procedures. We had obtained informed consent from patients prior to using these images. We also reviewed applicable sections of Appendix B prior to the colposcopy session and the LEEP session.

All learners were residents in obstetrics and gynecology. Before and after the simulation, learners completed questionnaires that inquired how comfortable they were in performing the following tasks:

1. Positioning and focusing the colposcope,
2. Identifying abnormal areas for biopsy during colposcopy,
3. Collecting cervical biopsies and performing endocervical curettage,
4. Assuring hemostasis after performance of colposcopy,
5. Understanding indications for LEEP,
6. Performing steps in preparing for the LEEP procedure,
7. Collecting the LEEP specimen, and
8. Assuring hemostasis after performance of LEEP.

We measured comfort level on a 5-point Likert scale (1 = *very uncomfortable,* 3 = *neither comfortable nor uncomfortable,* 5 = *very comfortable*). We then compared comfort levels before and after the simulation module using the Student *t* test. A *p* value of less than .05 was considered significant. Questionnaires also inquired about level of agreement with the following statements: “I feel prepared to perform colposcopies independently” and “I feel prepared to perform LEEPs independently.” We measured agreement level on a 5-point Likert scale (1 = *strongly disagree,* 3 = *neutral,* 5 = *strongly agree*). The following demographic data were also obtained: (1) year in residency and (2) number of colposcopies and LEEPs performed prior to simulation module. The questionnaires are in Appendix C.

### Equipment

The following equipment was used for successful implementation of the simulation case:

• Two female pelvic task trainers.
• Two colposcopes.
• Kielbasa sausage pieces measuring 3 inches in length with varying appearances signifying aceto-white changes and abnormal vasculature, which were created with correction fluid and red markers. The canal through the sausage also had to be created to simulate endocervix.
• Kielbasa sausage pieces measuring 6 inches in length for performance of LEEP.
• Two plastic specula.
• Two Kevorkian forceps for performance of biopsies at the time of colposcopy.
• Two endocervical curettes for performance of endocervical sampling at the time of colposcopy.
• Supplies to aid with visualization of cervix abnormalities: acetic acid, Lugol's solution.
• Supplies to aid with hemostasis: silver nitrate, ferrous subsulfate solution.
• Specimen containers.
• Return electrode pad.
• LEEP machine.
• LEEP handpiece.
• LEEP ball electrodes.
• LEEP loop electrodes.
• Two coated specula.
• 1% lidocaine with epinephrine.
• 22-gauge needle with 5-mL syringe.
• Spinal needle.
• Smoke evacuator and plastic tubing.
• Scopettes.
• Ring forceps.

### Personnel

All learners were residents in obstetrics and gynecology. The facilitators were attending gynecologists and fellows in gynecology (who had already completed a residency in obstetrics and gynecology). Two facilitators coordinated the colposcopy sessions. Two facilitators coordinated the LEEP sessions.

### Implementation

We performed this simulation module in two patient exam rooms and in a procedure room at the ambulatory gynecology center at Mount Sinai Hospital. Learners started with either the colposcopy session or the LEEP session. Each session lasted 1 hour. After completion of the first session, learners moved on to the second one. Key principles in the presimulation module reading were reviewed during the simulation sessions.

During the colposcopy session (held in patient examination rooms), sausages measuring 3 inches in length were placed in the pelvic task trainer. These sausage pieces had varying appearances of aceto-white changes and abnormal vasculature that were created with the use of correction fluid and a red marker. After placing the speculum in the pelvic task trainer to visualize the simulated cervix, learners used the colposcope to focus on the cervix. They then performed biopsies at aceto-white sites with Kevorkian forceps. Learners also had to demonstrate how to perform an endocervical sampling with the endocervical curette. They further had to demonstrate how they would attain hemostasis with either silver nitrate, ferrous subsulfate solution, or both. The endocervix was created by carving out a hole (with a knife) in the center of the sausage measuring less than 1 cm in diameter and approximately 1 cm in length.

During the LEEP session, sausages measuring 6 inches in length, with return electrode pad attached to the back of the sausage, were placed in the pelvic exam trainer. Learners placed the coated speculum into the pelvic exam trainer and visualized the cervix. Learners then had to apply Lugol's solution to the sausage. Learners also had to perform the steps of LEEP, which were as follows:

1. Filling the syringe with lidocaine and injecting it at correct sites of the cervix with the spinal needle;
2. Threading tubing attached to the smoke evacuator into the coated speculum;
3. Performing the excisional procedure with the loop;
4. Removing the excised portion of the cervix with ring forceps;
5. Performing endocervical sampling with the endocervical curette;
6. Using the ball electrode to simulate cauterization of areas noted to be bleeding, taking care to avoid excessive cautery that could lead to cervical stenosis; and
7. Demonstrating attainment of hemostasis with silver nitrate, ferrous subsulfate, or both.

### Results

Twenty-one resident learners participated in the simulation sessions held in November 2021, and 13 participated in May 2022. Table 1 outlines comfort levels before and after participation in the simulation module pertaining to performance of the following: identifying abnormal areas for biopsy during colposcopy, collecting cervical biopsies and performing endocervical curettage, assuring hemostasis after performance of colposcopy, understanding indications for LEEP, preparing for LEEP, collecting the LEEP specimen, and assuring hemostasis after performance of LEEP. Mean learner comfort levels for all parameters above (except understanding indications for LEEP) significantly increased after involvement in the project (*p* < .01).

**Table 1.**
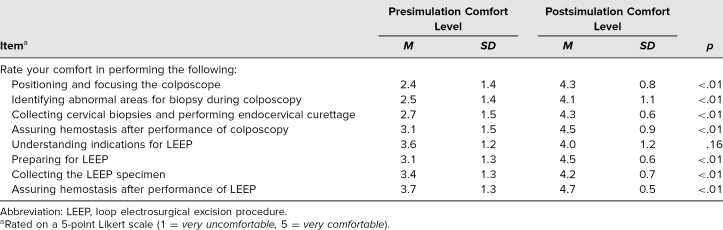
Comfort Levels Before and After Simulation (*N* = 34)

Table 2 outlines degree of agreement with the following statements: “I feel prepared to perform colposcopies independently” and “I feel prepared to perform LEEPs independently.” Mean learner agreement pertaining to colposcopy significantly increased from 2.2 to 3.5 (*p* < .01). Mean learner agreement pertaining to LEEP also increased (2.9 to 3.6); statistical difference approached significance at .06. These results did not vary over postgraduate year level. These statistical differences were not different from those on analyses performed separately for each of the two sets of sessions.

**Table 2.**
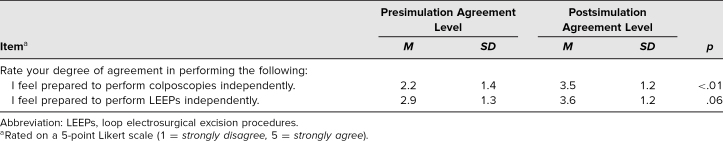
Agreement With Statements Before and After Simulation (*N* = 34)

On feedback obtained at the end of the sessions, 15 out of the 17 learners who provided feedback in November in 2021 and seven out of the eight who provided feedback in May 2022 indicated that the sessions were very helpful and that they would like to participate in these sessions again in the future. All other comments received were favorable.

## Discussion

This simulation-based module was created to refine resident performance of colposcopy and LEEP. The simulation modules were conducted in November 2021 and May 2022. The frequency at which these modules took place was dependent on scheduling of other resident didactics.

Comfort level pertaining to performance of the steps associated with colposcopy and LEEP significantly improved after participation in the simulation sessions. Learner confidence in ability to perform colposcopy and LEEP independently also increased. Across both modules, 88% of learners found the sessions to be very helpful.

Although learner confidence in the ability to perform LEEP increased after participation in the simulation sessions, this difference was not as significant as that relating to learner confidence in the ability to perform colposcopy independently. We anticipate that learners were less likely to be confident in performing LEEP given the greater number of steps needed to perform it successfully than to perform colposcopy. These findings did not vary across postgraduate year level. Our findings attest to the importance of improving learner comfort prior to performing these procedures in the clinical setting and assuring that adequate time and explanations are provided at the time of training sessions.

One of our session's limitations is the focus on comfort with specific steps of performance of colposcopy and LEEP as opposed to assessing for correct performance after the training sessions. This would be the basis for a future study idea utilizing the schemes that have been presented here. A related limitation is the absence of data on retention of skills over time. This could be the rationale for another study, which would be key in further establishing the utility of this simulation curriculum. A third limitation is absence of improvement in knowledge on performance of colposcopy before and after LEEP. An additional study intervention in the future could involve allotting time during the modules to review procedure indications and also giving learners pre- and postmodule questionnaires assessing knowledge pertaining to these procedures. Nonetheless, we were able to overcome some of the obstacles encountered in conducting these modules, such as obtaining supplies (which fortunately was done through departmental funding) and coordinating instructor and learning schedules. A fourth limitation of our study is absence of a formal course evaluation as there were fewer than five sessions. Based on informal feedback, the sessions were very well received, and all learners felt that that the sausage model was effective in teaching them how to perform the LEEP. This is in agreement both with our team's assessment on the efficacy of a sausage model in teaching this skill during the preparation for the sessions and with that in the literature. Walters and colleagues found that the sausage model aided in improving resident knowledge, skills, and confidence regarding performance of LEEP.^7^ A limitation of the sausage model pertains to colposcopy and demonstrating aceto-white changes for which we had to use correction fluid to mimic the range of aceto-white changes one would see with cervical dysplasia.

In conclusion, we present commendable data in support of the idea that a simulation-based module on performance of colposcopy and LEEP significantly increases learner comfort in the performance of this type of procedure. This simulation session can be held in a variety of locations and is not time-intensive. Moreover, it can be implemented with reusable equipment.

## Appendices


Facilitators Guide.docxColposcopy LEEP Didactics.pptxQuestionnaires.docx
*All appendices are peer reviewed as integral parts of the Original Publication.*
