# Supplementary material for: Colposcopy and Loop Electrosurgical Excision Procedure: A Simulated Exercise
Source: MedEdPORTAL. 2023 Sep 8;19:11344. doi: 10.15766/mep_2374-8265.11344 (PMC10485179; doi:10.15766/mep_2374-8265.11344)
Supplement: Supplementary file 1 — Facilitators Guide.docxColposcopy LEEP Didactics.pptxQuestionnaires.docx [file mep_2374-8265.11344-s001.zip › C. Questionnaires.docx]

**APPENDIX C: Questionnaires**

**Pre-Simulation Questionnaire**

1. **Please enter the number of your home address followed by the second digit of your age (to create a unique identifier. i.e. If you live at 245 North Ave and are 32 years old, enter 2452)**
2. **Please select your year in residency:**

PGY-1

PGY-2

PGY-3

PGY-4

1. **How many COLPOSCOPIES have you performed in the past?**

0

1-5

6-10

11+

**Questions 4 and 5 refer to COLPOSCOPIES.**

1. **How comfortable do you feel with each of the following?**

|  | Very uncomfortable | Somewhat uncomfortable | Neither comfortable nor uncomfortable | Somewhat comfortable | Very comfortable |
| --- | --- | --- | --- | --- | --- |
| Positioning and focusing the colposcope |  |  |  |  |  |
| Identifying abnormal areas for biopsy during colposcopy |  |  |  |  |  |
| Collecting cervical biopsies and endocervical curettage |  |  |  |  |  |
| Assuring hemostasis |  |  |  |  |  |

1. **Please indicate how much you agree/disagree with the following statement.**

|  | Strongly disagree | Disagree | Neutral | Agree | Strongly agree |
| --- | --- | --- | --- | --- | --- |
| I feel prepared to perform colposcopy independently |  |  |  |  |  |

1. **How many LEEPs have you performed in the past?**

0

1-5

6-10

11+

**Questions 7 and 8 refer to LEEPs.**

1. **How comfortable do you feel with each of the following?**

|  | Very uncomfortable | Somewhat uncomfortable | Neither comfortable nor uncomfortable | Somewhat comfortable | Very comfortable |
| --- | --- | --- | --- | --- | --- |
| Understanding indications for LEEP |  |  |  |  |  |
| Steps in preparing for  LEEP procedure |  |  |  |  |  |
| Collecting the LEEP specimen |  |  |  |  |  |
| Assuring hemostasis |  |  |  |  |  |

1. **Please indicate how much you agree/disagree with the following statement.**

|  | Strongly disagree | Disagree | Neutral | Agree | Strongly agree |
| --- | --- | --- | --- | --- | --- |
| I feel prepared to perform LEEPs independently |  |  |  |  |  |

**Post-Simulation Questionnaire**

1. **Please enter the number of your home address followed by the second digit of your age (to create a unique identifier. i.e. If you live at 245 North Ave and are 32 years old, enter 2452)**

**Questions 2 and 3 refer to COLPOSCOPIES.**

1. **How comfortable you do feel with each of the following?**

|  | Very uncomfortable | Somewhat uncomfortable | Neither comfortable nor uncomfortable | Somewhat comfortable | Very comfortable |
| --- | --- | --- | --- | --- | --- |
| Positioning and focusing the colposcope |  |  |  |  |  |
| Identifying abnormal areas for biopsy during colposcopy |  |  |  |  |  |
| Collecting cervical biopsies and endocervical curettage |  |  |  |  |  |
| Assuring hemostasis |  |  |  |  |  |

1. **Please indicate how much you agree/disagree with the following statement.**

|  | Strongly disagree | Disagree | Neutral | Agree | Strongly agree |
| --- | --- | --- | --- | --- | --- |
| I feel prepared to perform colposcopy independently |  |  |  |  |  |

**Questions 4 and 5 refer to LEEPs.**

1. **How comfortable do you feel with each of the following?**

|  | Very uncomfortable | Somewhat uncomfortable | Neither comfortable nor uncomfortable | Somewhat comfortable | Very comfortable |
| --- | --- | --- | --- | --- | --- |
| Understanding indications for LEEP |  |  |  |  |  |
| Steps in preparing for  LEEP procedure |  |  |  |  |  |
| Collecting the LEEP specimen |  |  |  |  |  |
| Assuring hemostasis |  |  |  |  |  |

1. **Please indicate how much you agree/disagree with the following statement.**

|  | Strongly disagree | Disagree | Neutral | Agree | Strongly agree |
| --- | --- | --- | --- | --- | --- |
| I feel prepared to perform LEEPs independently |  |  |  |  |  |

1. **How helpful was this simulation didactic overall?**

Very helpful Somewhat helpful Neither helpful nor helpful Somewhat unhelpful

1. **I felt the time allotted for this simulation was...**

Too long Too short Just right
