# Supplementary material for: Colposcopy and Loop Electrosurgical Excision Procedure: A Simulated Exercise
Source: MedEdPORTAL. 2023 Sep 8;19:11344. doi: 10.15766/mep_2374-8265.11344 (PMC10485179; doi:10.15766/mep_2374-8265.11344)
Supplement: Supplementary file 1 — Facilitators Guide.docxColposcopy LEEP Didactics.pptxQuestionnaires.docx [file mep_2374-8265.11344-s001.zip › A. Facilitators Guide.docx]

**Colposcopy and Loop Electrosurgical Excision Procedure (LEEP) Simulation: Facilitator’s Guide**

All images are author-owned.

*Educational Objectives*

- Identify indications for colposcopy and LEEP
- Position and focus the colposcope
- Collect cervical biopsies of abnormal areas and perform endocervical curettage
- Prepare for LEEP and collect the LEEP specimen
- Identify methods for assuring hemostasis after performance of colposcopy and LEEP

All learners should receive and review Appendix B one week prior to the scheduled session.

Learners are to complete questionnaires at the start of the module and at the end.

*Setup of rooms – 1 hour*

1. Three rooms are to be used: two for colposcopy, one for loop electrosurgical excision procedure (LEEP).
2. Colposcopy room should have the following:
   - Colposcope attached to video monitor
   - Table with necessary equipment
   - Patient exam table with pelvic exam trainer placed on it
   - Pelvic exam task trainer should have piece of sausage placed through opening in back
   - Pelvic exam task trainer should have plastic speculum placed in it
   - Sausages should be cut to three inches in length. Hole should be cut in center to resemble cervical os. White out and red markers should be used to simulate aceto-white changes.


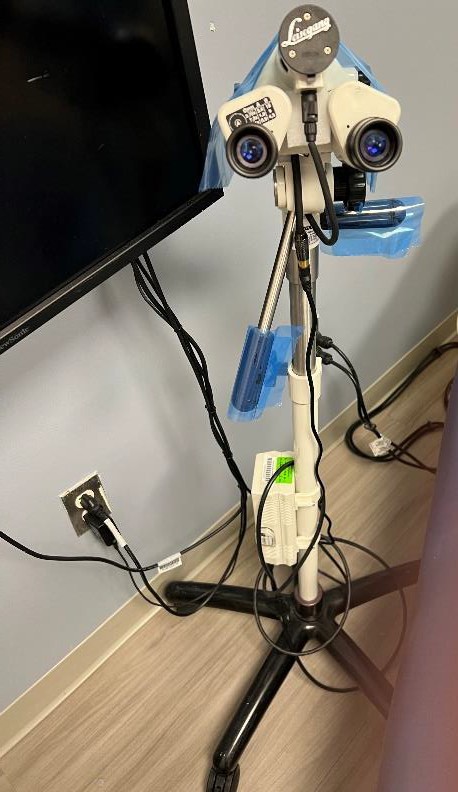


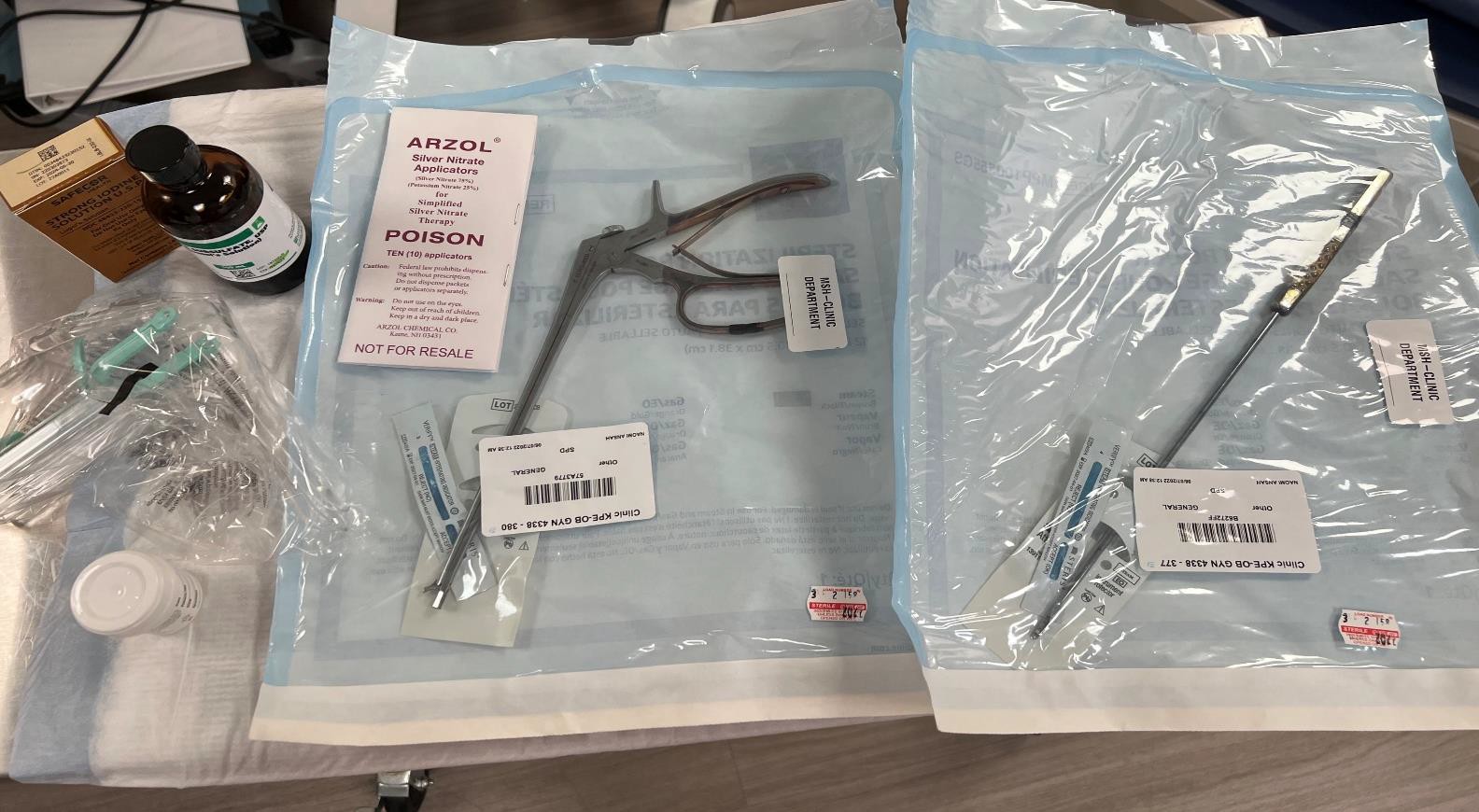


Lugol’s solution

Specimen container

Endocervical curette

Plastic speculum

Kevorkian forceps

Silver nitrate

Ferrous subulfate

Colposcope Selected colposcopy equipment


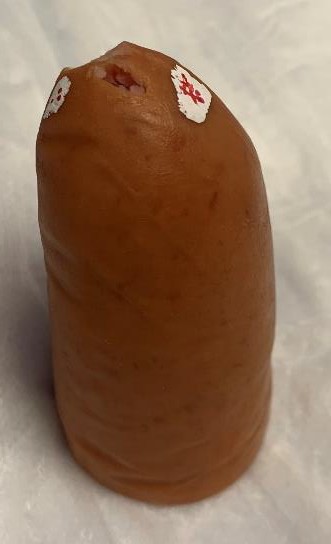

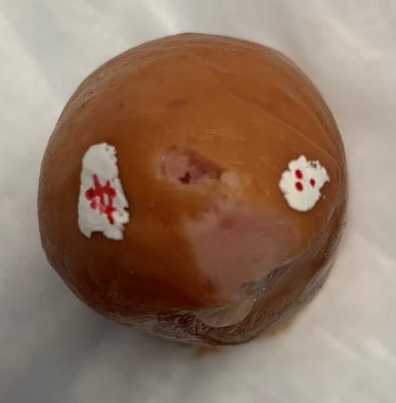

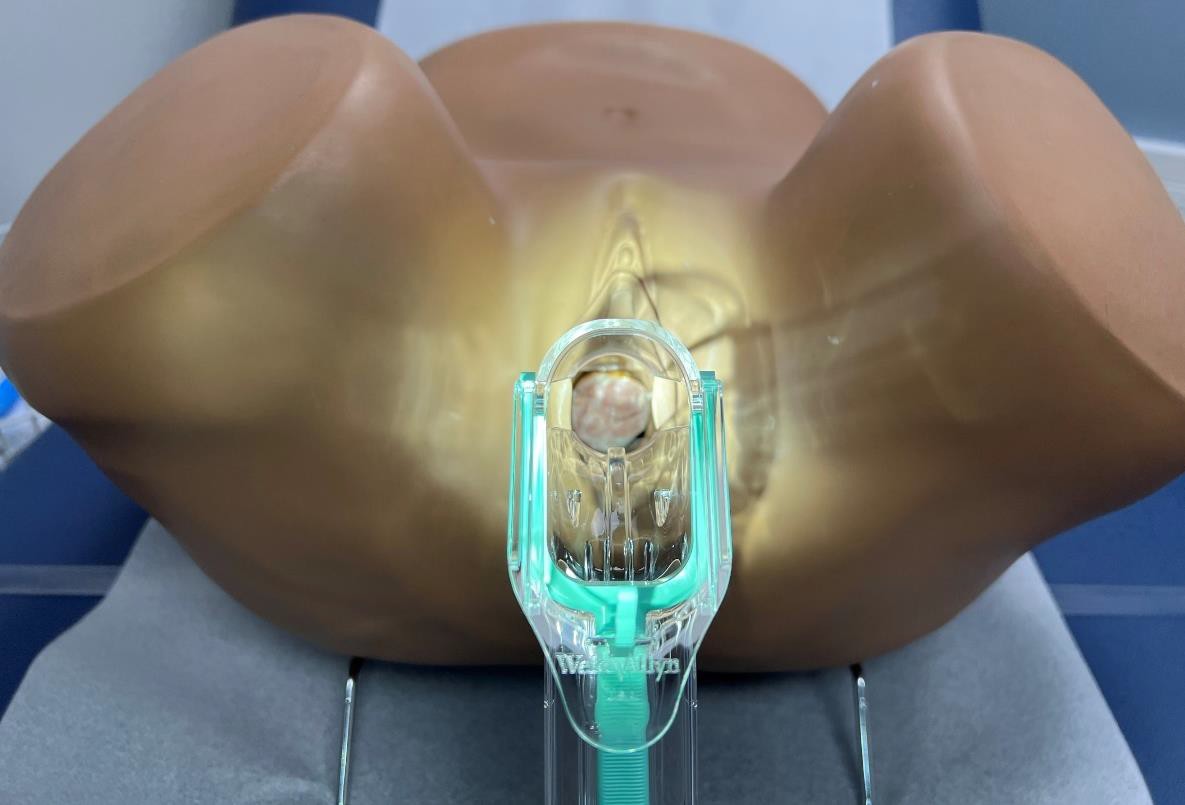


Back

Front

Front

Colposcopy; plastic speculum in task trainer Sausages for colposcopy

1. LEEP room should have the following:
   - LEEP machine
   - Table with necessary equipment
   - Patient exam table with pelvic exam trainer placed on it
   - Pelvic exam task trainer should have piece of sausage placed through opening in back
   - Sausages should be cut to six inches in length. Hole should be cut in center to resemble cervical os. Sausage should have return electrode pad wrapped around it
   - Pelvic exam task trainer should have coated speculum placed in it with tubing threaded through it. Tubing should be attached to smoke evacuator which is to be placed in LEEP machine.


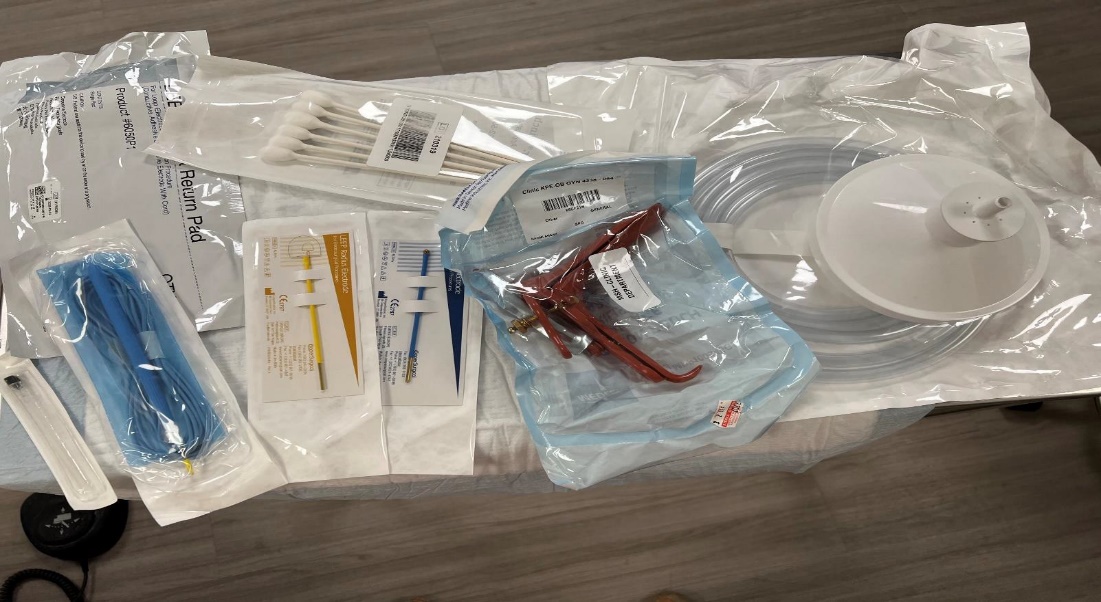

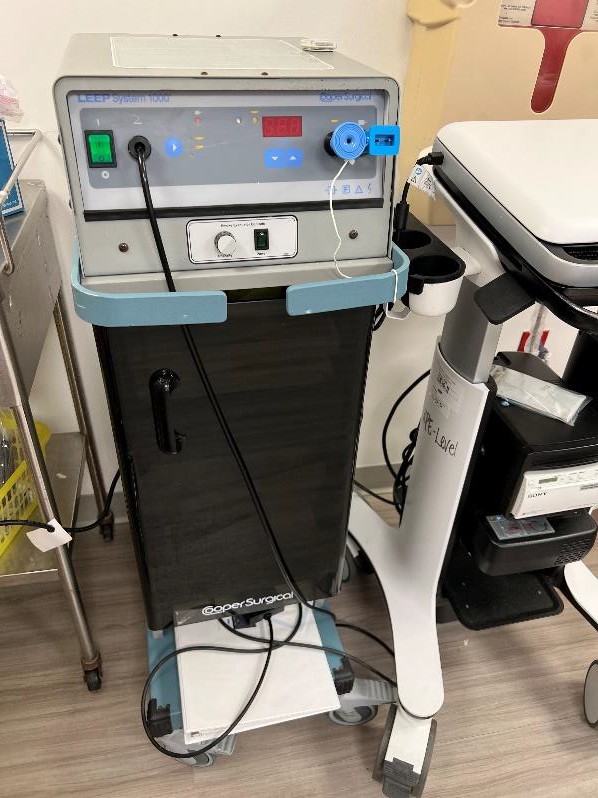


Return electrode pad

Smoke evacuator with tubing

Coated speculum

LEEP electrode

Spinal needle

LEEP rollerball

LEEP loop

Scopettes

LEEP machine including pedal Selected LEEP equipment


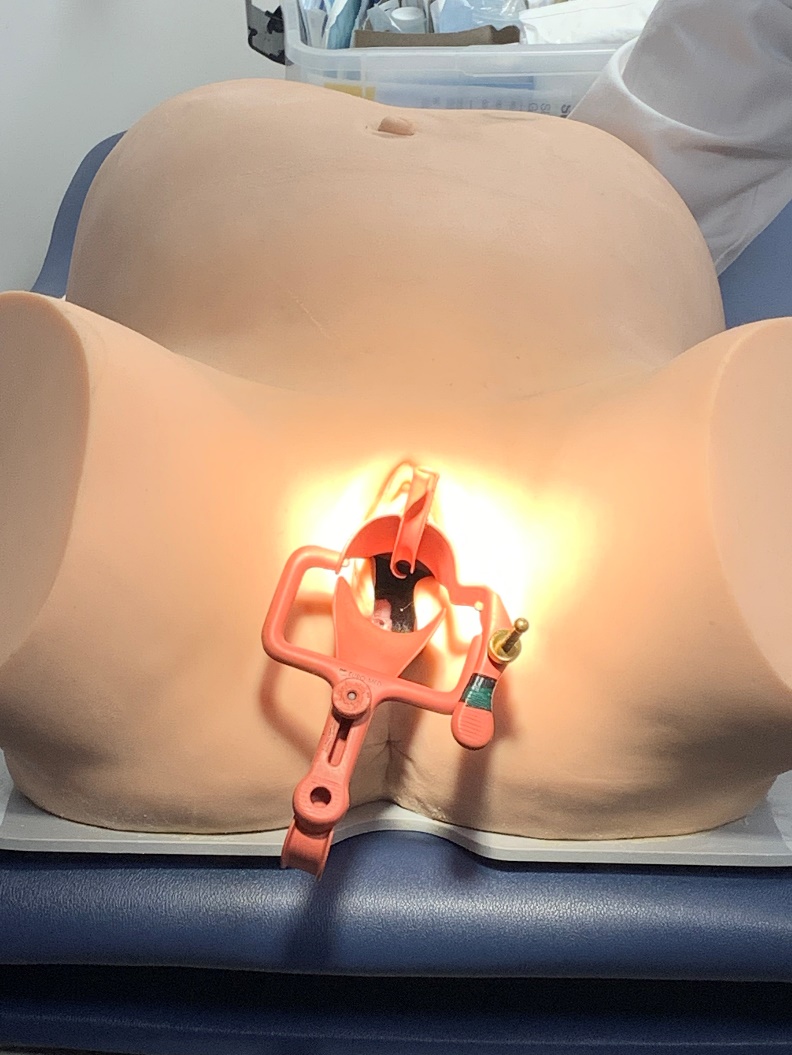
 *Sausage for LEEP*

Back

Front


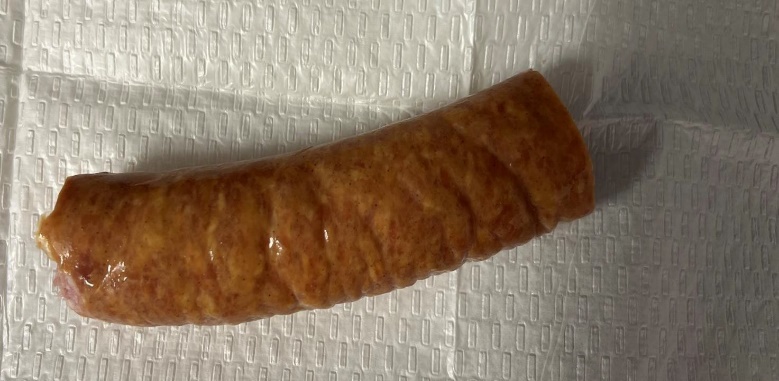


Return electrode pad

LEEP; coated speculum in task trainer

*Colposcopy Session (60 minutes in duration)* - Maximum participants: 12

1. 10 minutes should be spent to review indications for colposcopy and for facilitator to review how to perform and how to use all tools on tray. Facilitator should review content in slide numbers 5-32 of Appendix B.
2. Each participant will demonstrate the following on the simulated pelvic exam trainer and cervix (“sausage”):
   - Insertion of plastic speculum and visualization of cervix.
   - Cleaning of cervix with scopette.
   - Application of acetic acid to cervix after placing scopette in acetic acid
   - Performance of biopsies at “aceto-white” sites with Kevorkian forceps; bottom blade is to be placed inside cervical os (“opening in sausage”) and blades are to then be brought together to remove piece of cervix (“sausage”).
   - Performance of endocervical curettage with curette through cervical os (“opening in sausage”).
   - Application of silver nitrate stick and ferrous subsulfate to “aceto-white” sites to simulate how to attain hemostasis.
   - Placement of biopsy specimens (“sausage pieces”) in specimen containers.

*LEEP Session (60 minutes in duration)* - Maximum participants: 12

1. 10 minutes should be spent to review indications for LEEP and for facilitator to review how to perform and how to use all tools on tray. Facilitator should review content in slide numbers 33-37 of Appendix B.
2. Each participant will demonstrate the following on the simulated pelvic exam trainer and cervix (“sausage”):
   - Insertion of coated speculum and visualization of cervix.
   - Threading of plastic tubing (attached to smoke evacuator) through coated speculum.
   - Adjustment of settings for performance of LEEP on machine (50 Watts on “CUT,” 50 Watts on “COAG”).
   - Application of Lugol’s solution.
   - Injection of lidocaine at the following sites on simulated cervix: 3 o’clock, 9 o’clock, 12 o’clock
   - Performance of LEEP with loop after attaching loop to LEEP electrode.
   - Performance of endocervical curettage with curette through cervical os (“opening in sausage”).
   - Demonstration of cauterization at base of excised cervix with rollerball after attaching to rollerball to LEEP electrode.
   - Application of silver nitrate stick and ferrous subsulfate to base of excised cervix to simulate how to attain hemostasis.
   - Placement of specimens (“sausage pieces”) in specimen containers.
