# Supplementary material for: Colposcopy and Loop Electrosurgical Excision Procedure: A Simulated Exercise
Source: MedEdPORTAL. 2023 Sep 8;19:11344. doi: 10.15766/mep_2374-8265.11344 (PMC10485179; doi:10.15766/mep_2374-8265.11344)
Supplement: Supplementary file 1 — Facilitators Guide.docxColposcopy LEEP Didactics.pptxQuestionnaires.docx [file mep_2374-8265.11344-s001.zip › B. Colposcopy LEEP Didactics.pptx]

## Slide 1
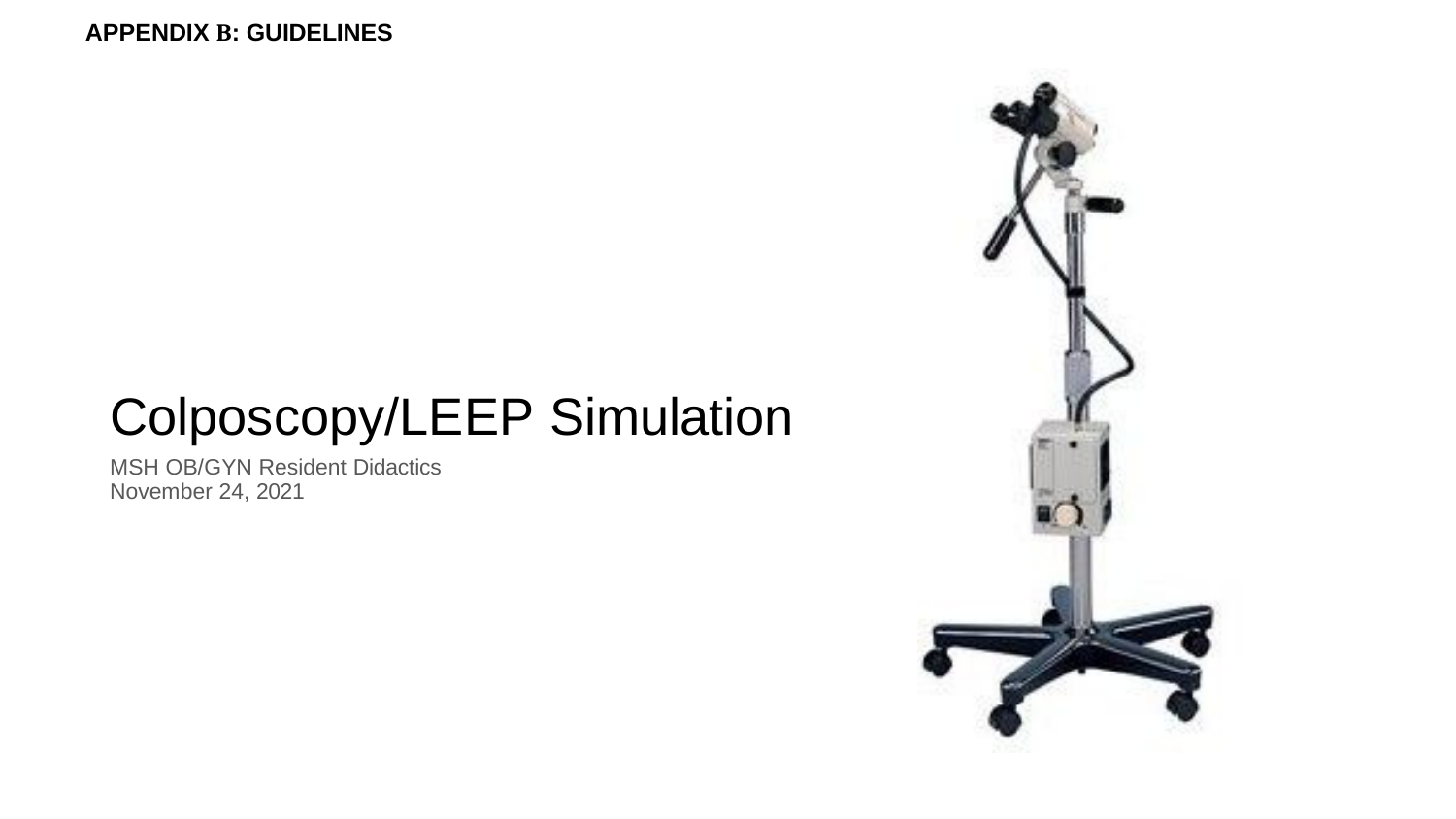

APPENDIX B: GUIDELINES
Colposcopy/LEEP Simulation
MSH OB/GYN Resident Didactics November 24, 2021

## Slide 2
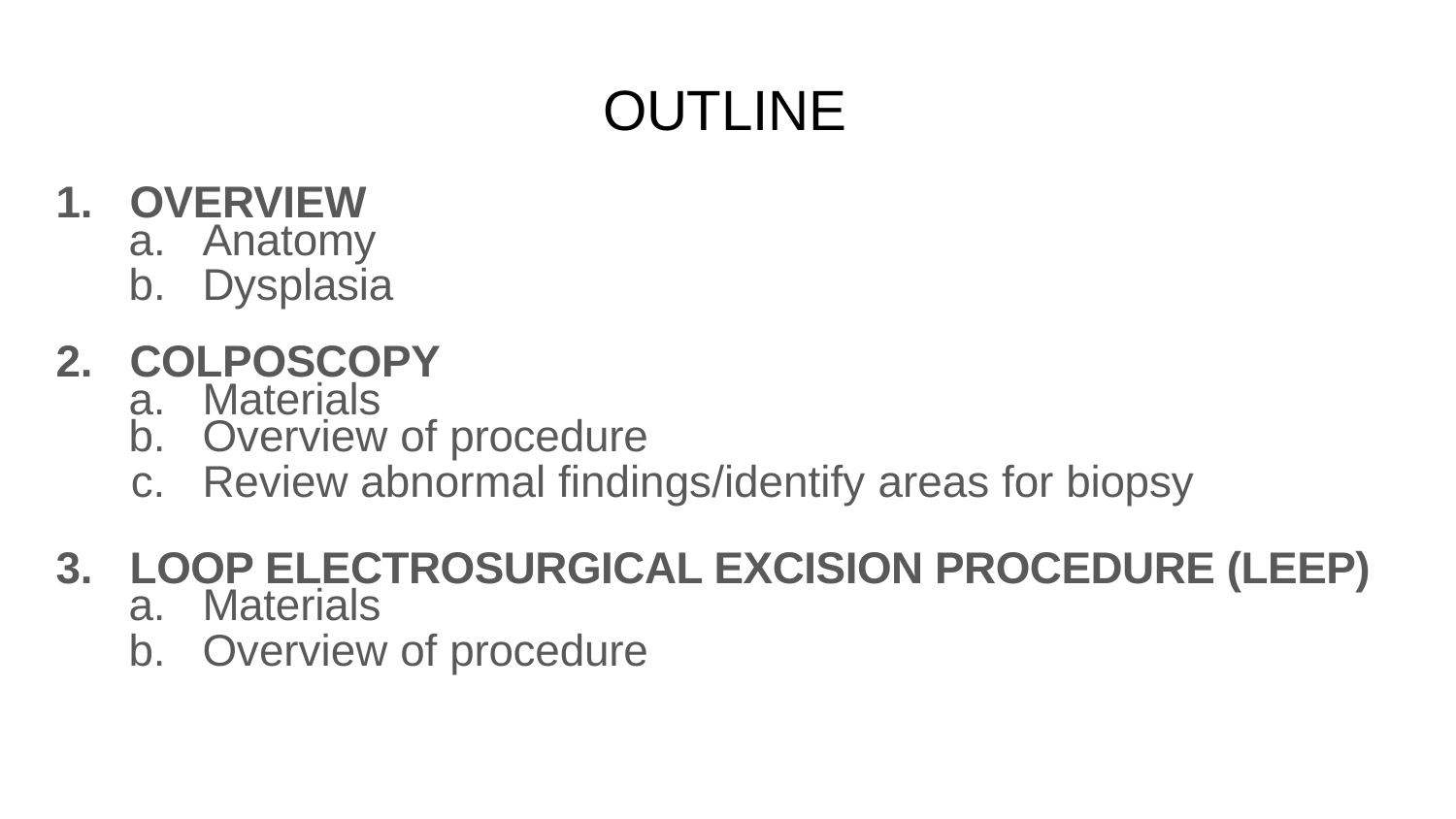

# OUTLINE
OVERVIEW
Anatomy
Dysplasia
COLPOSCOPY
Materials
Overview of procedure
Review abnormal findings/identify areas for biopsy
LOOP ELECTROSURGICAL EXCISION PROCEDURE (LEEP)
Materials
Overview of procedure

## Slide 3
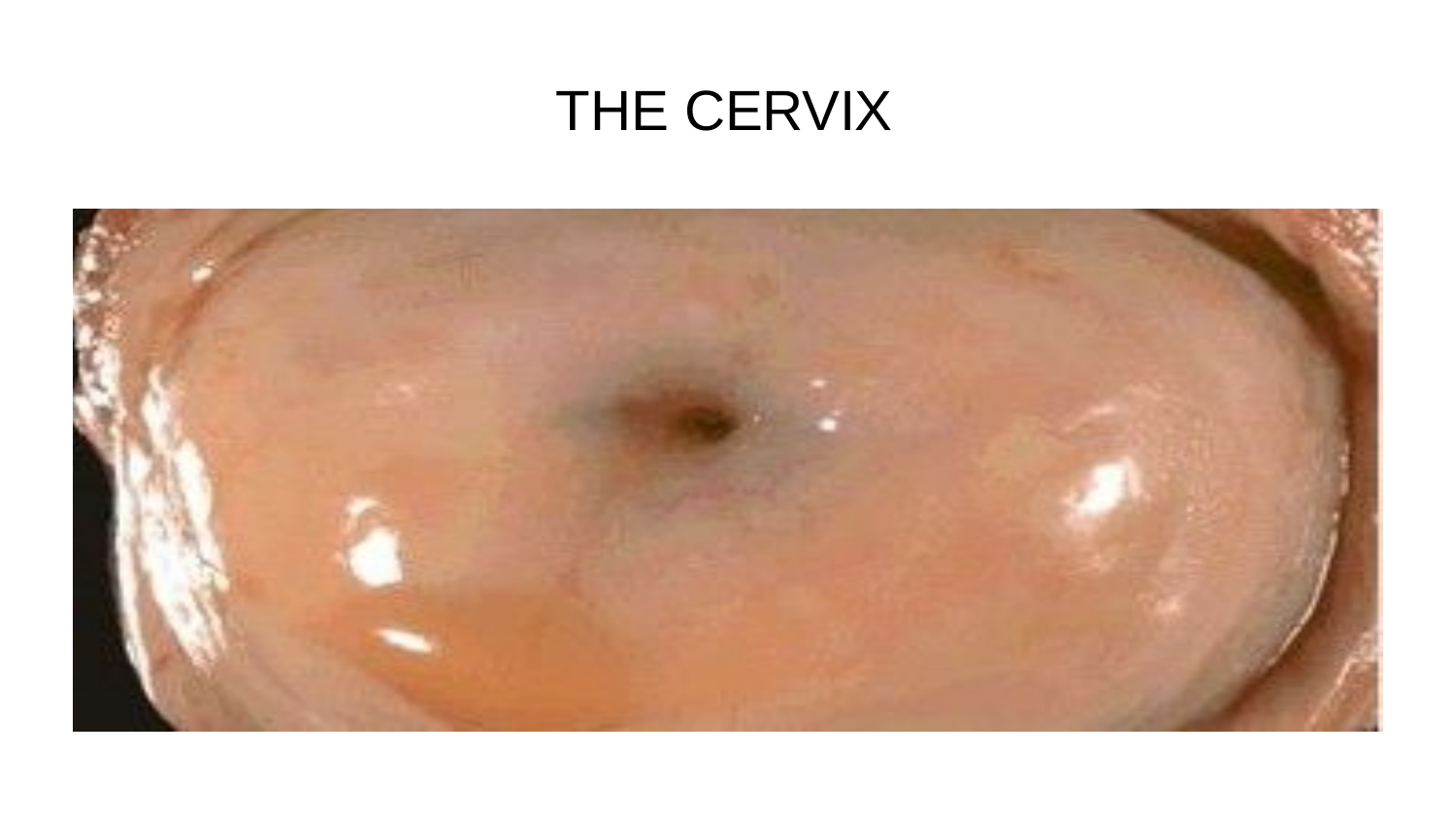

# THE CERVIX

## Slide 4
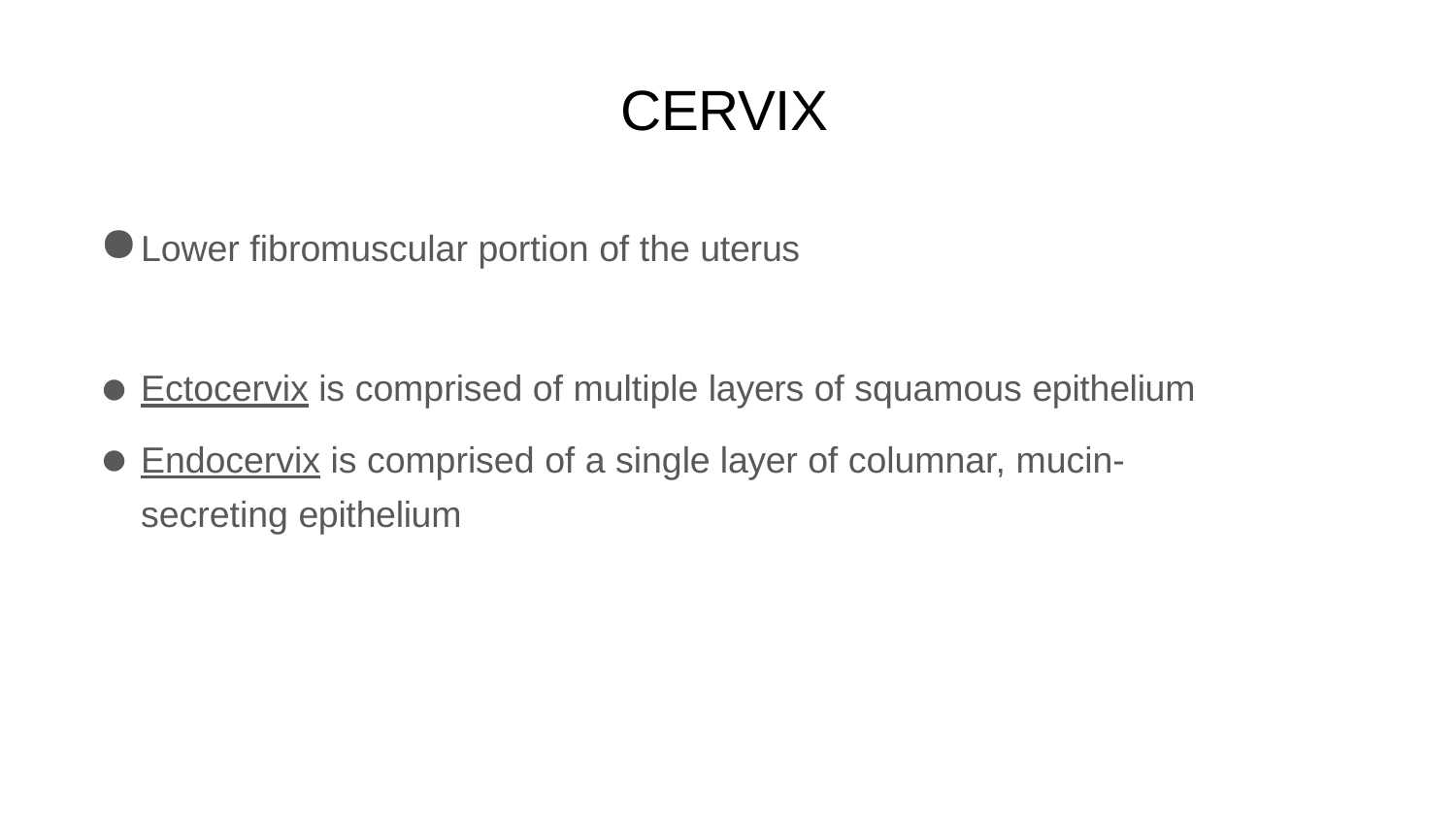

# CERVIX
Lower fibromuscular portion of the uterus
Ectocervix is comprised of multiple layers of squamous epithelium
Endocervix is comprised of a single layer of columnar, mucin-secreting epithelium

## Slide 5
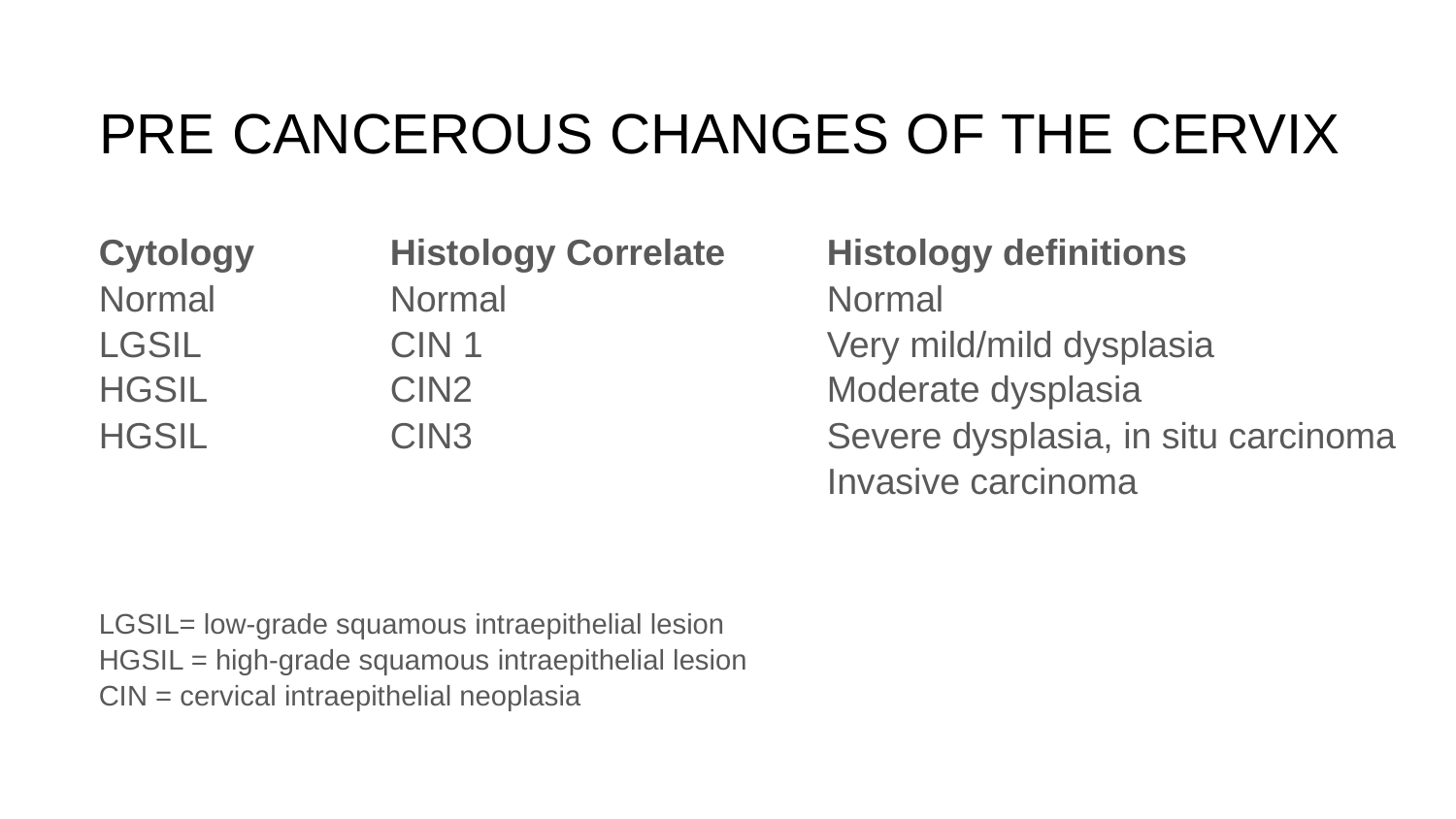

# PRE CANCEROUS CHANGES OF THE CERVIX
Cytology	Histology Correlate	Histology definitions
Normal		Normal			Normal
LGSIL		CIN 1			Very mild/mild dysplasia
HGSIL		CIN2			Moderate dysplasia
HGSIL		CIN3			Severe dysplasia, in situ carcinoma
						Invasive carcinoma
LGSIL= low-grade squamous intraepithelial lesion
HGSIL = high-grade squamous intraepithelial lesion
CIN = cervical intraepithelial neoplasia

## Slide 6
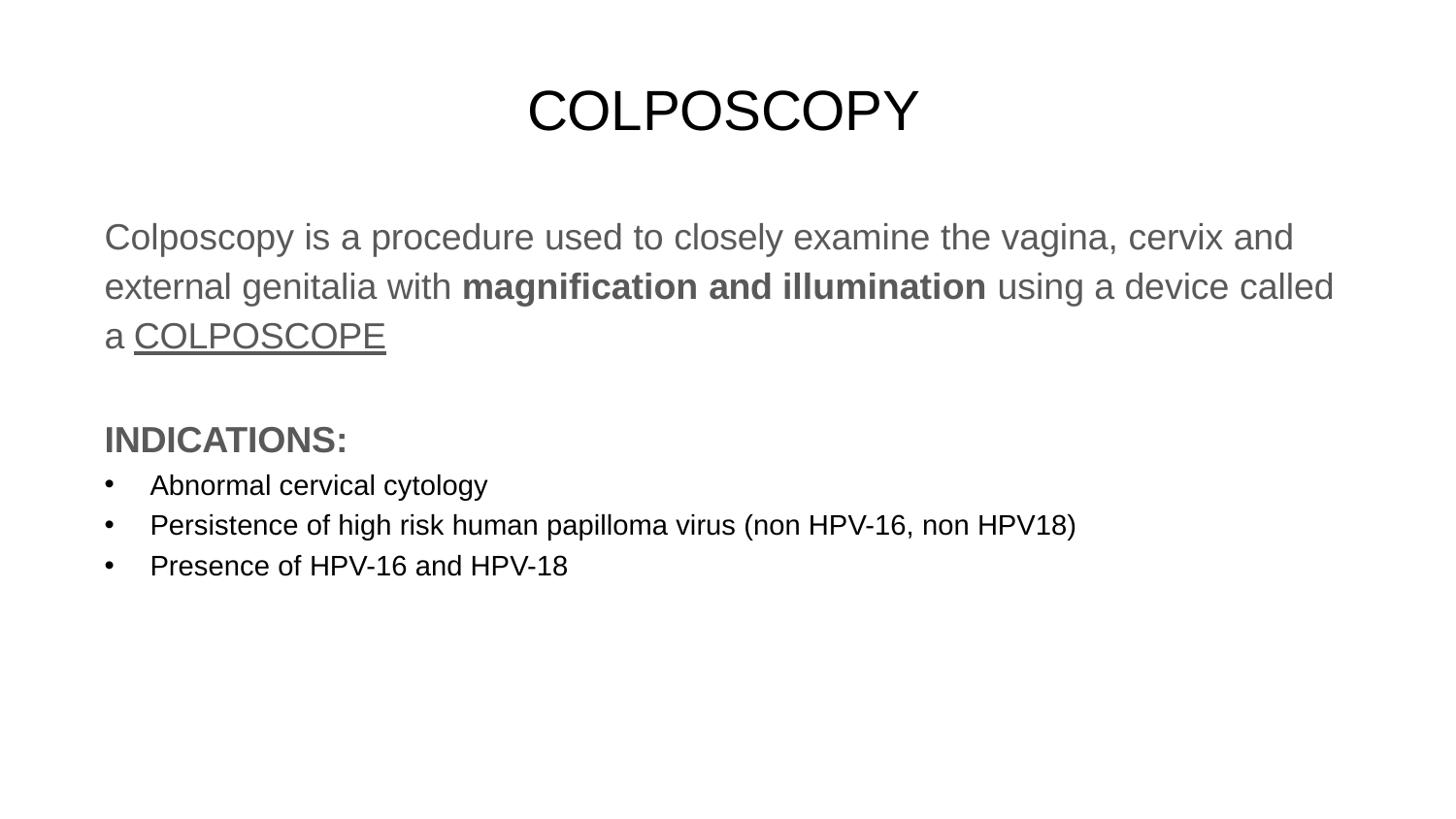

# COLPOSCOPY
Colposcopy is a procedure used to closely examine the vagina, cervix and external genitalia with magnification and illumination using a device called a COLPOSCOPE
INDICATIONS:
Abnormal cervical cytology
Persistence of high risk human papilloma virus (non HPV-16, non HPV18)
Presence of HPV-16 and HPV-18

## Slide 7
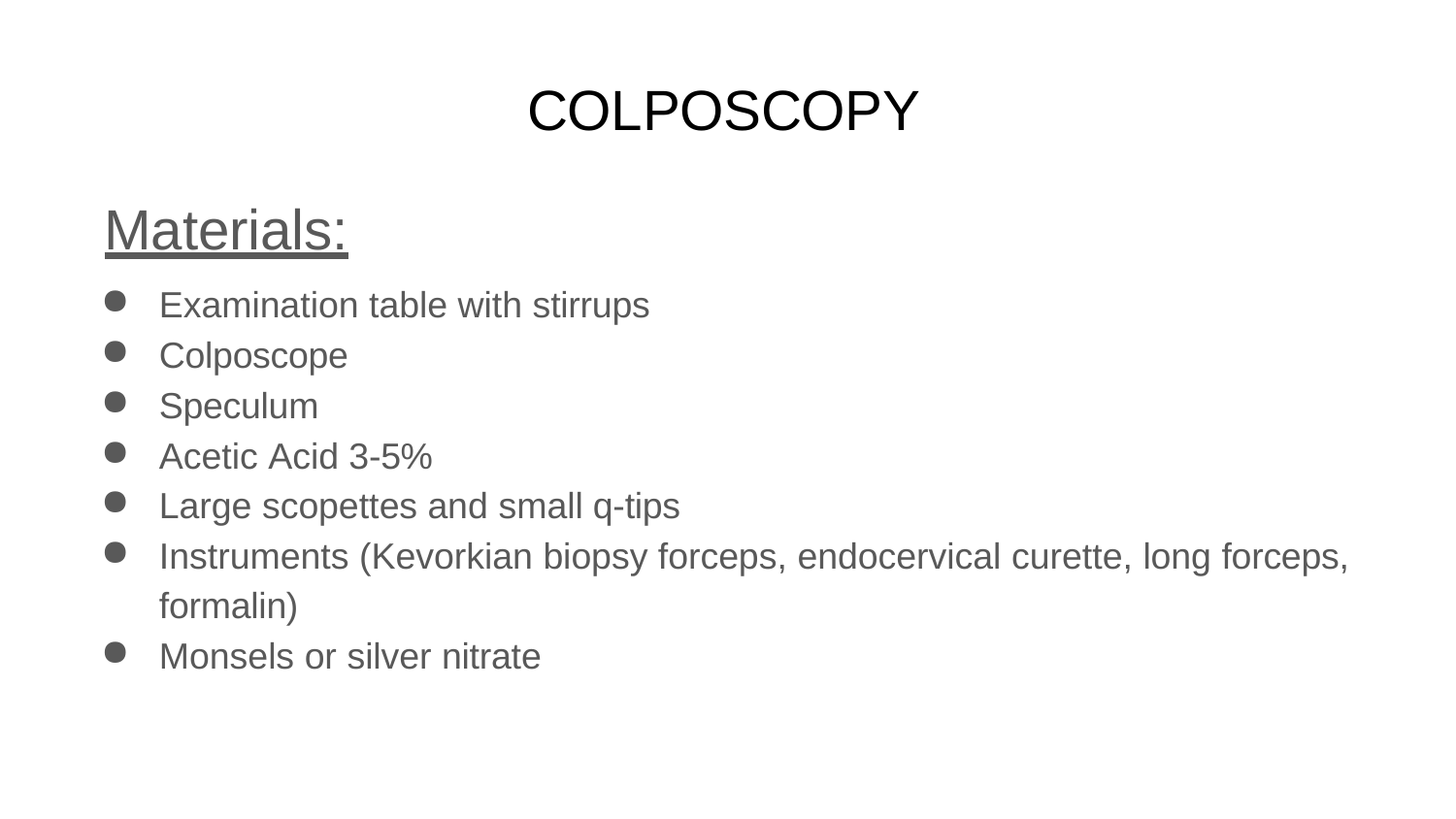

# COLPOSCOPY
Materials:
Examination table with stirrups
Colposcope
Speculum
Acetic Acid 3-5%
Large scopettes and small q-tips
Instruments (Kevorkian biopsy forceps, endocervical curette, long forceps, formalin)
Monsels or silver nitrate

## Slide 8
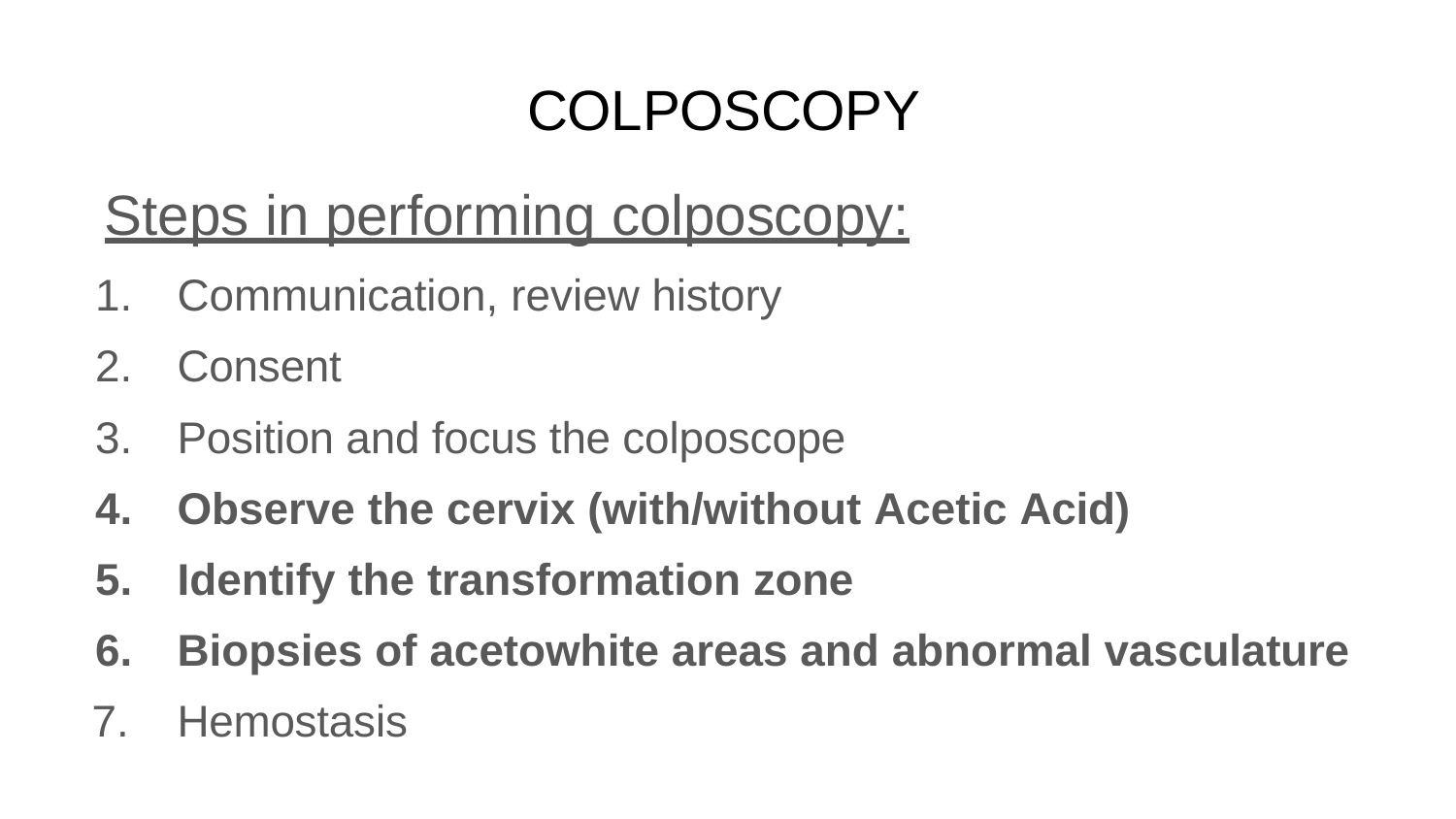

# COLPOSCOPY
Steps in performing colposcopy:
Communication, review history
Consent
Position and focus the colposcope
Observe the cervix (with/without Acetic Acid)
Identify the transformation zone
Biopsies of acetowhite areas and abnormal vasculature
Hemostasis

## Slide 9
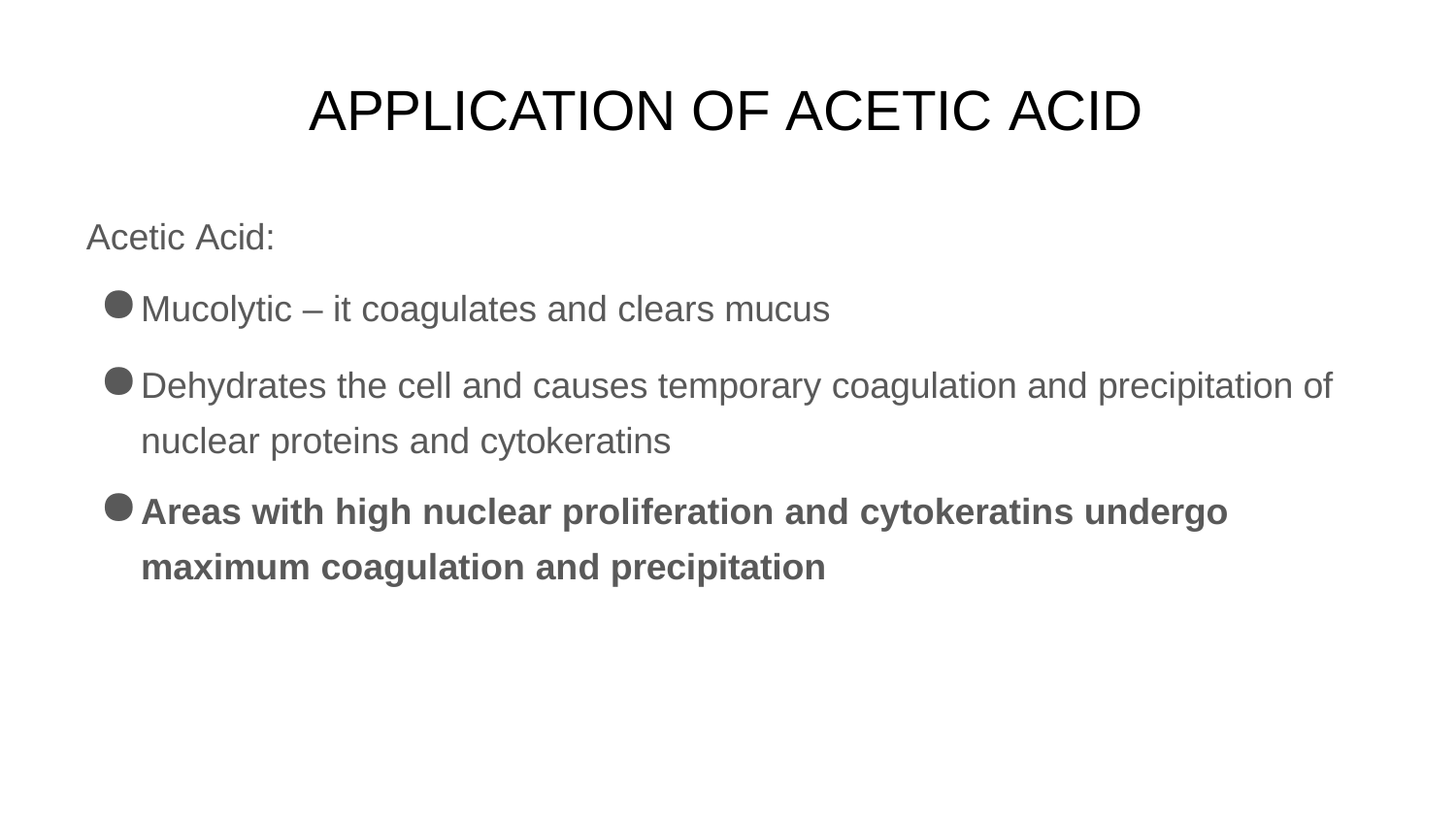

# APPLICATION OF ACETIC ACID
Acetic Acid:
Mucolytic – it coagulates and clears mucus
Dehydrates the cell and causes temporary coagulation and precipitation of nuclear proteins and cytokeratins
Areas with high nuclear proliferation and cytokeratins undergo maximum coagulation and precipitation

## Slide 10
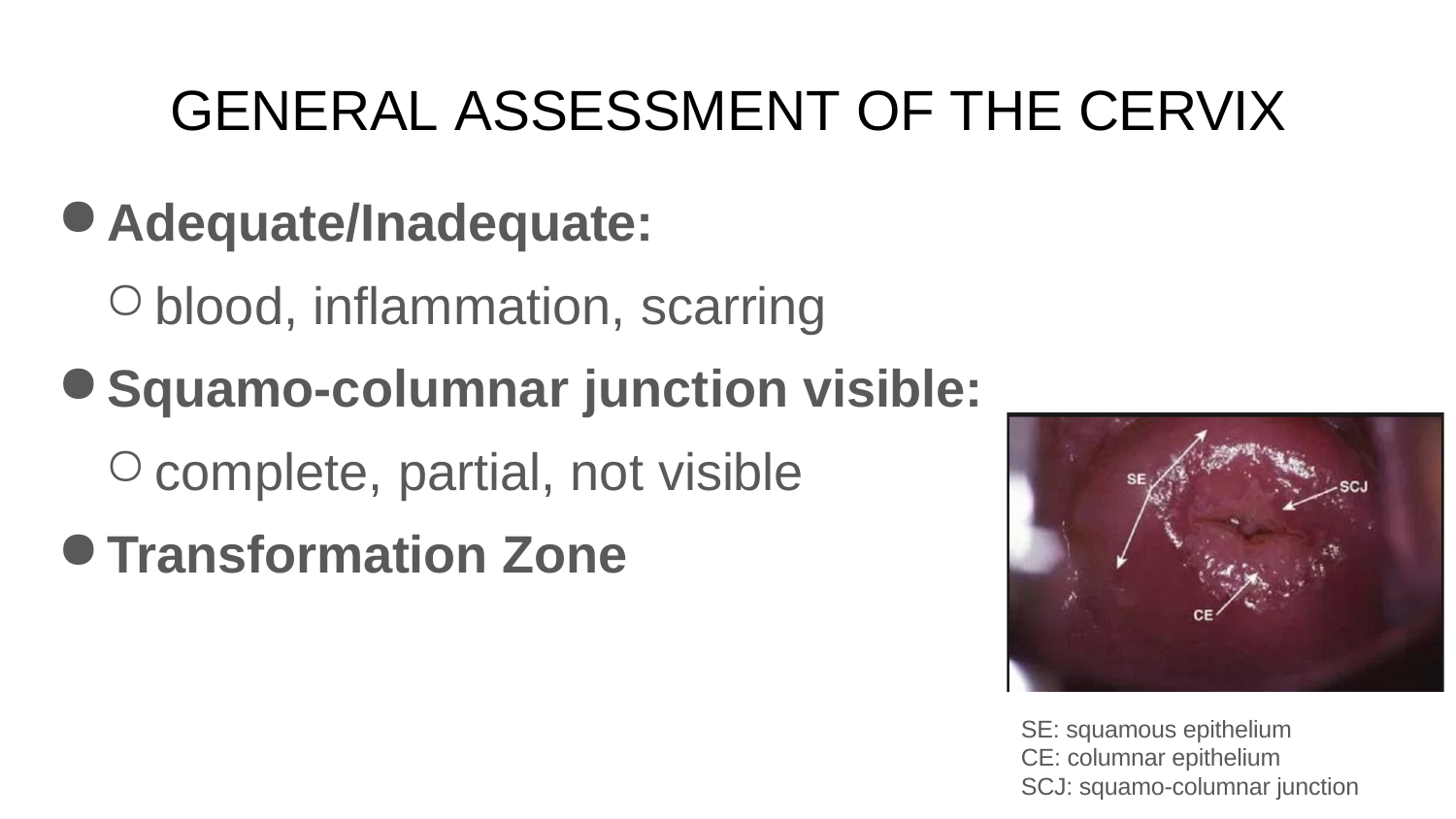

# GENERAL ASSESSMENT OF THE CERVIX
Adequate/Inadequate:
blood, inflammation, scarring
Squamo-columnar junction visible:
complete, partial, not visible
Transformation Zone
SE: squamous epithelium
CE: columnar epithelium
SCJ: squamo-columnar junction

## Slide 11
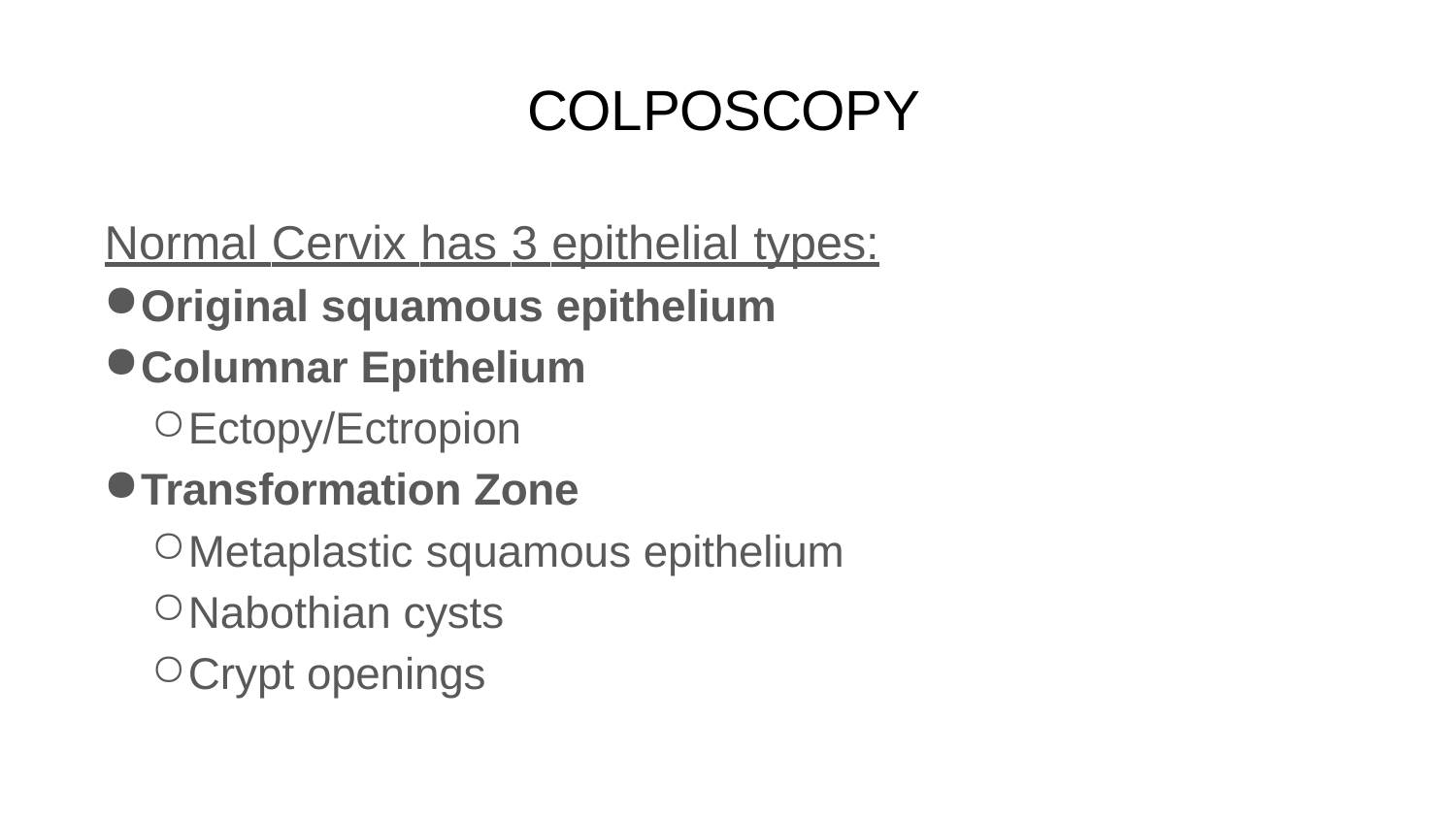

# COLPOSCOPY
Normal Cervix has 3 epithelial types:
Original squamous epithelium
Columnar Epithelium
Ectopy/Ectropion
Transformation Zone
Metaplastic squamous epithelium
Nabothian cysts
Crypt openings

## Slide 12
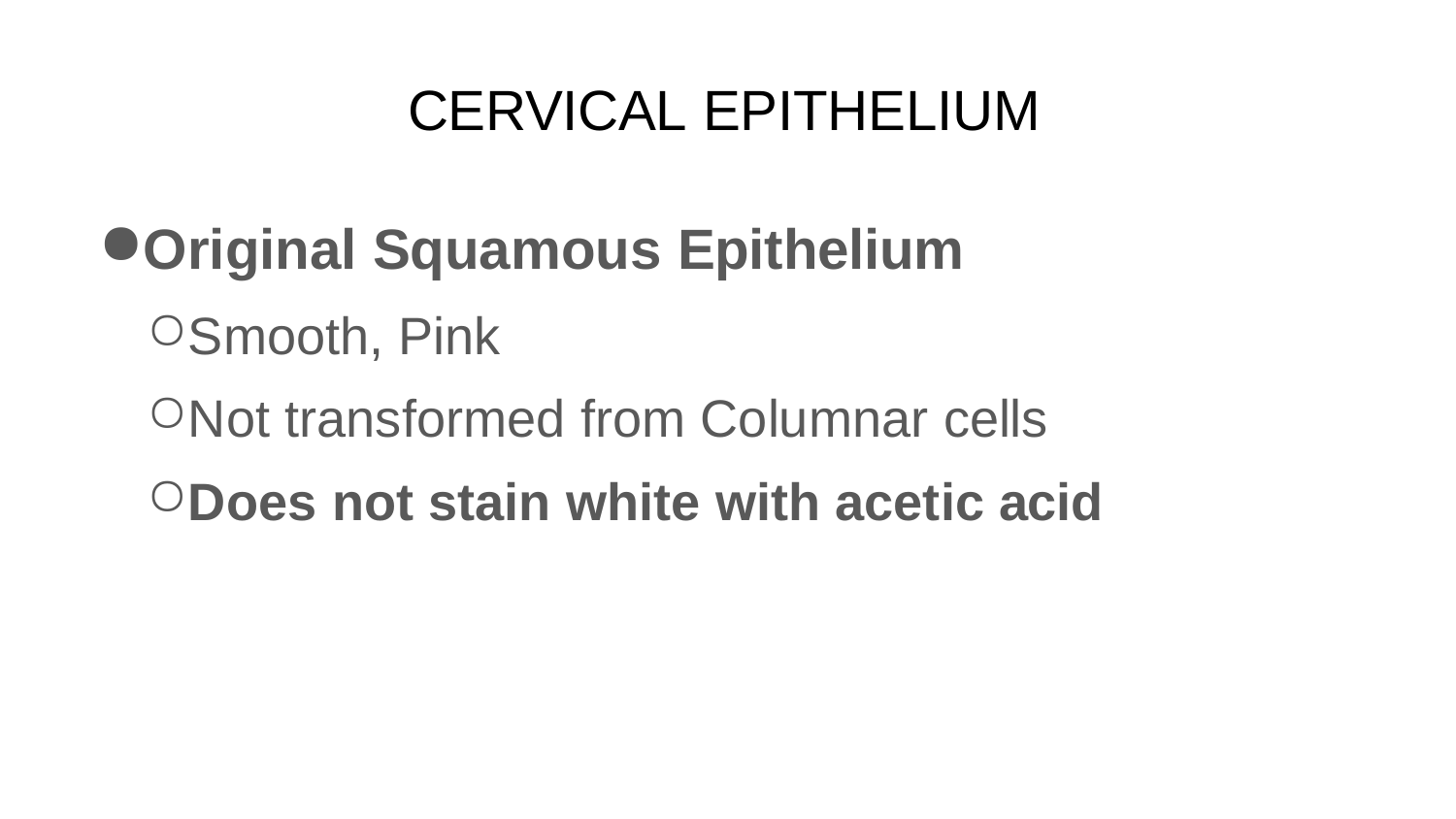

# CERVICAL EPITHELIUM
Original Squamous Epithelium
Smooth, Pink
Not transformed from Columnar cells
Does not stain white with acetic acid

## Slide 13
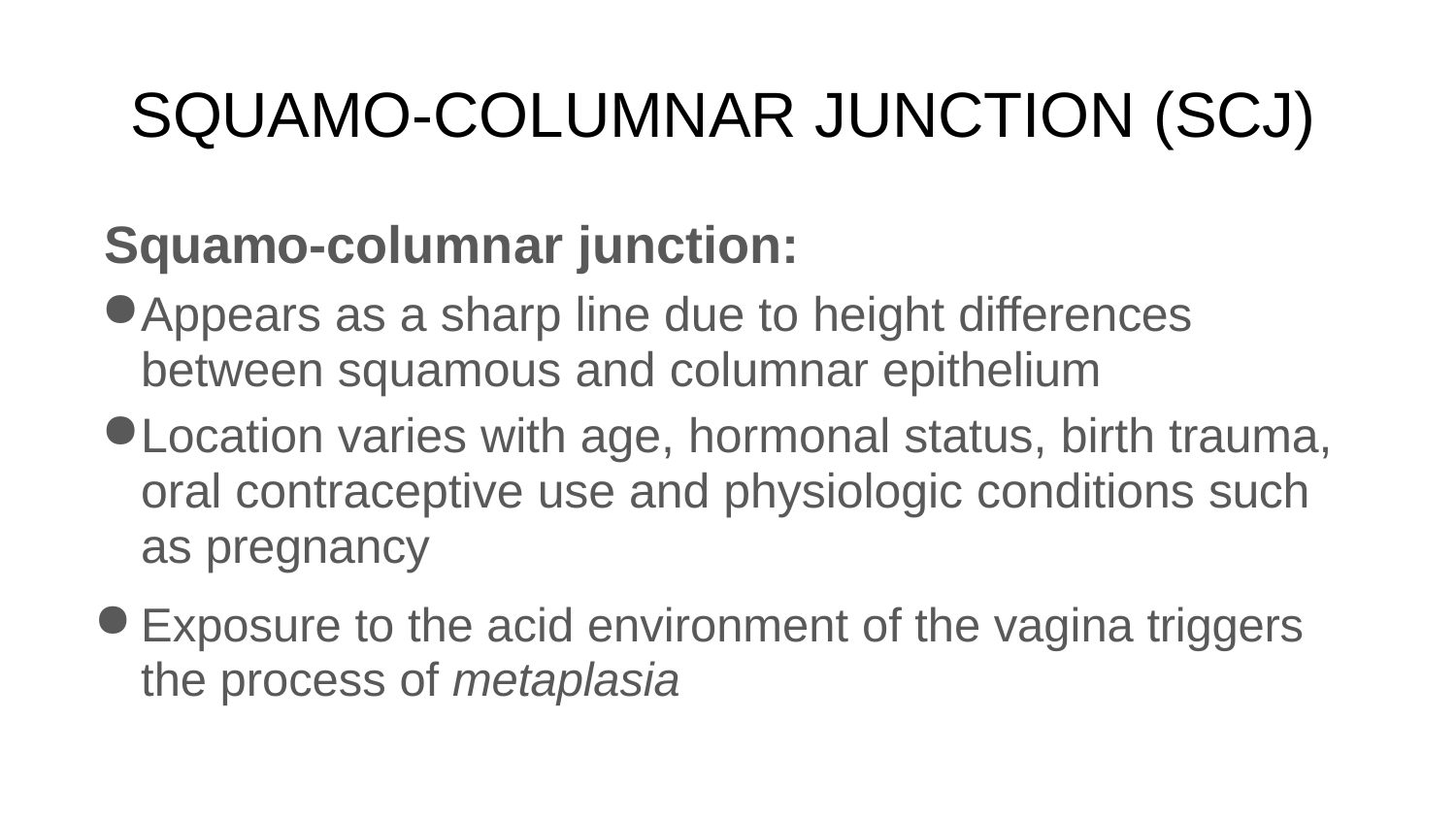

# SQUAMO-COLUMNAR JUNCTION (SCJ)
Squamo-columnar junction:
Appears as a sharp line due to height differences between squamous and columnar epithelium
Location varies with age, hormonal status, birth trauma, oral contraceptive use and physiologic conditions such as pregnancy
Exposure to the acid environment of the vagina triggers the process of metaplasia

## Slide 14
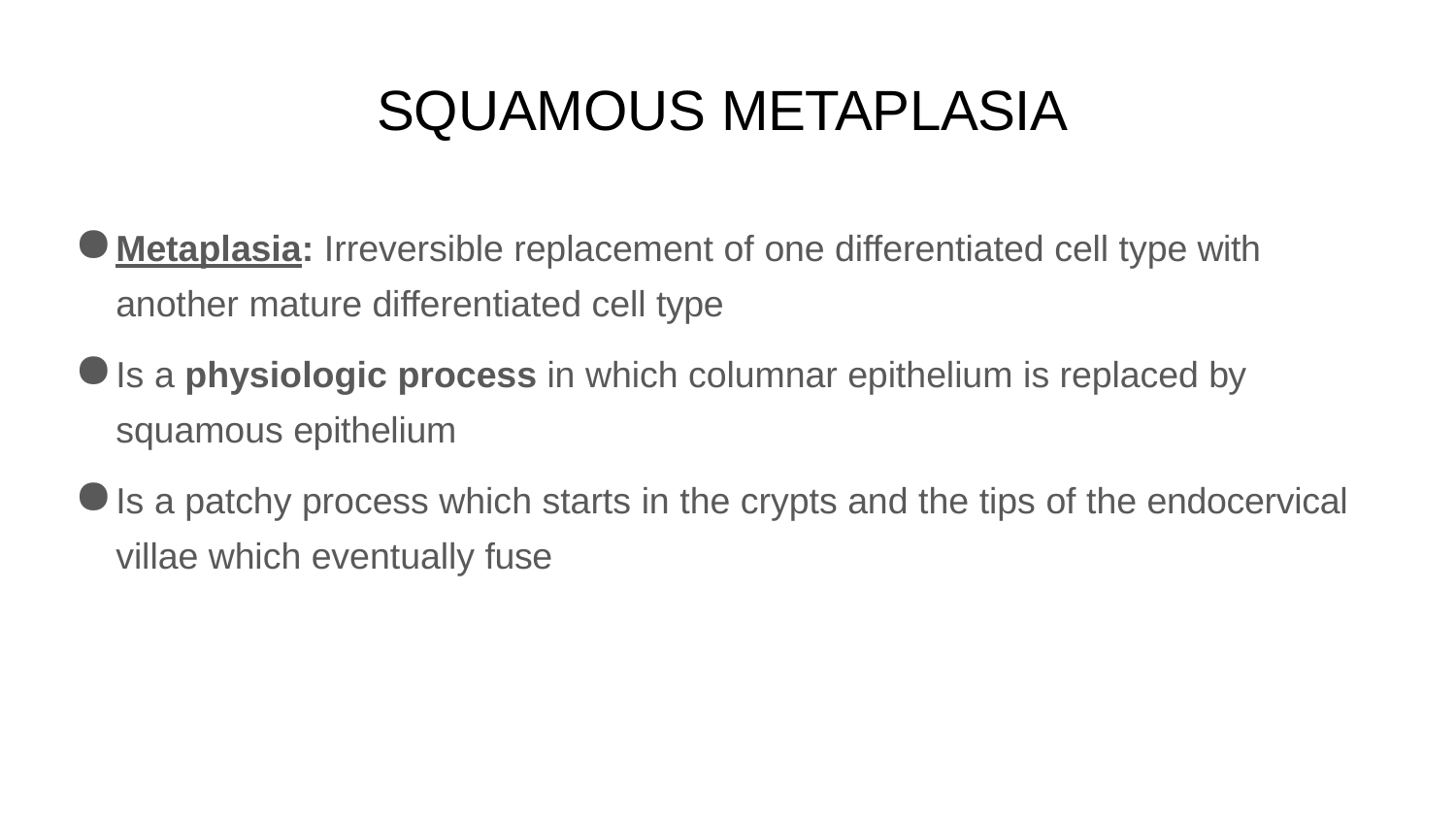

# SQUAMOUS METAPLASIA
Metaplasia: Irreversible replacement of one differentiated cell type with another mature differentiated cell type
Is a physiologic process in which columnar epithelium is replaced by squamous epithelium
Is a patchy process which starts in the crypts and the tips of the endocervical villae which eventually fuse

## Slide 15
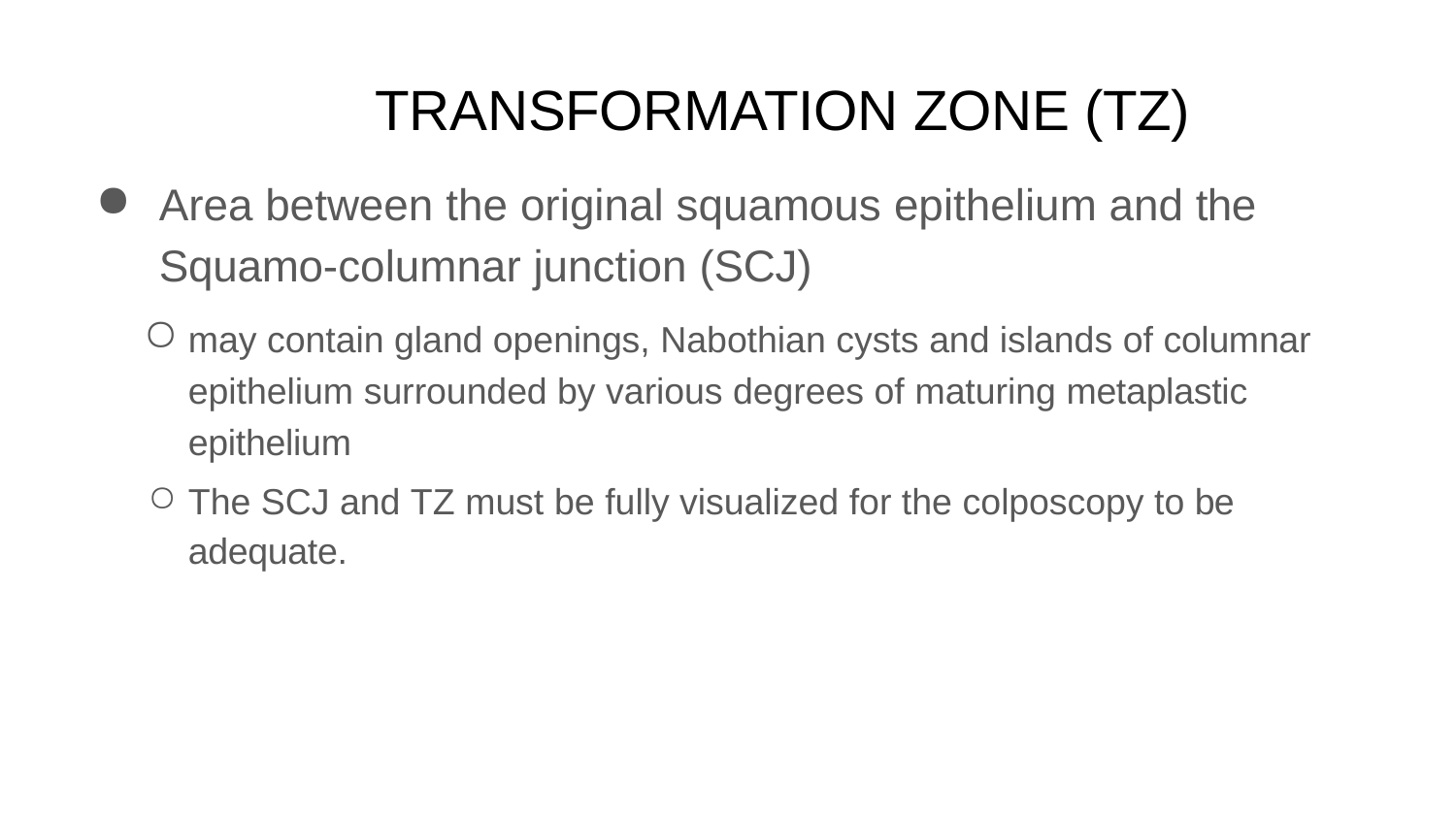

# TRANSFORMATION ZONE (TZ)
Area between the original squamous epithelium and the Squamo-columnar junction (SCJ)
may contain gland openings, Nabothian cysts and islands of columnar epithelium surrounded by various degrees of maturing metaplastic epithelium
The SCJ and TZ must be fully visualized for the colposcopy to be adequate.

## Slide 16
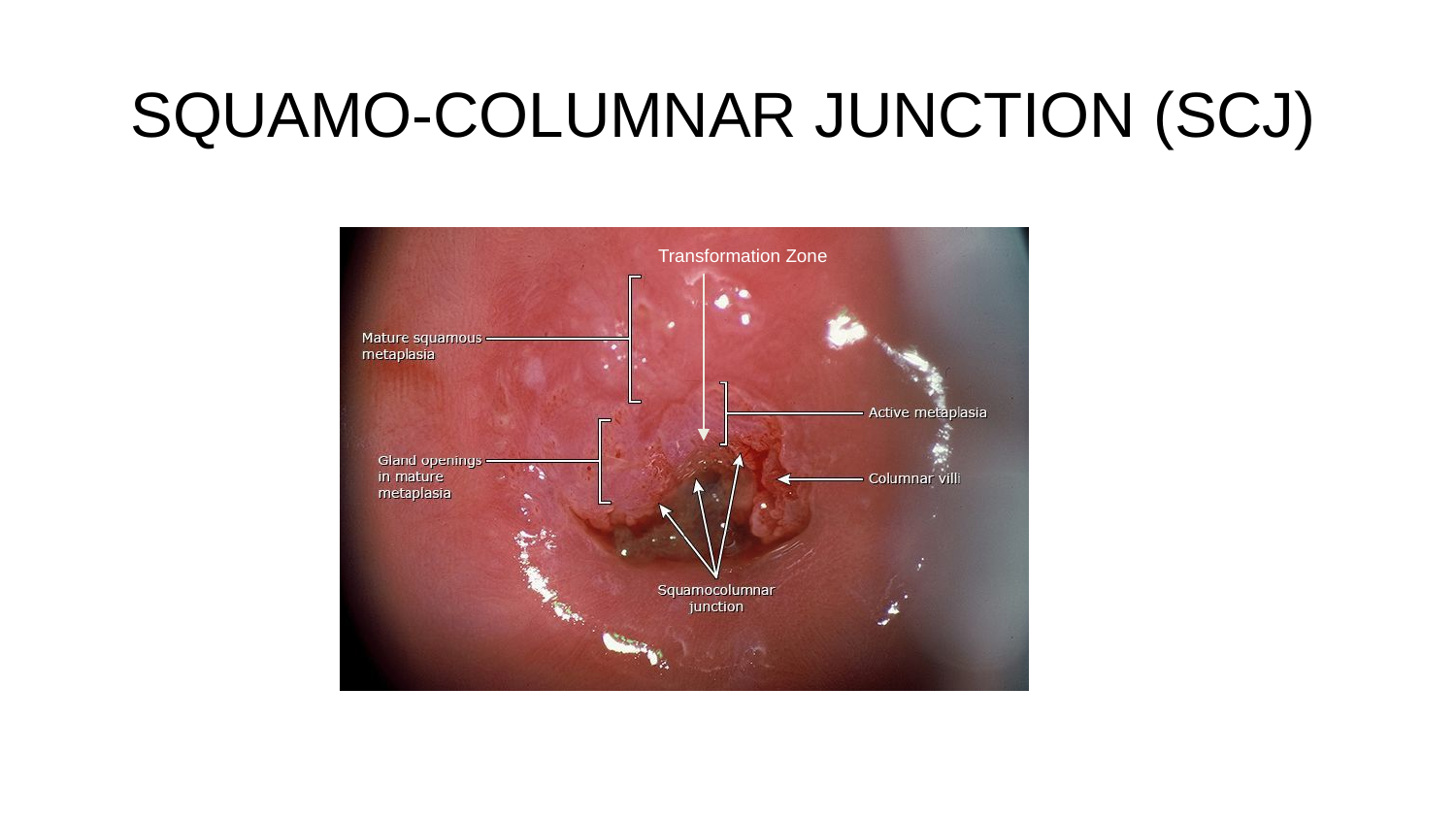

# SQUAMO-COLUMNAR JUNCTION (SCJ)
Transformation Zone

## Slide 17
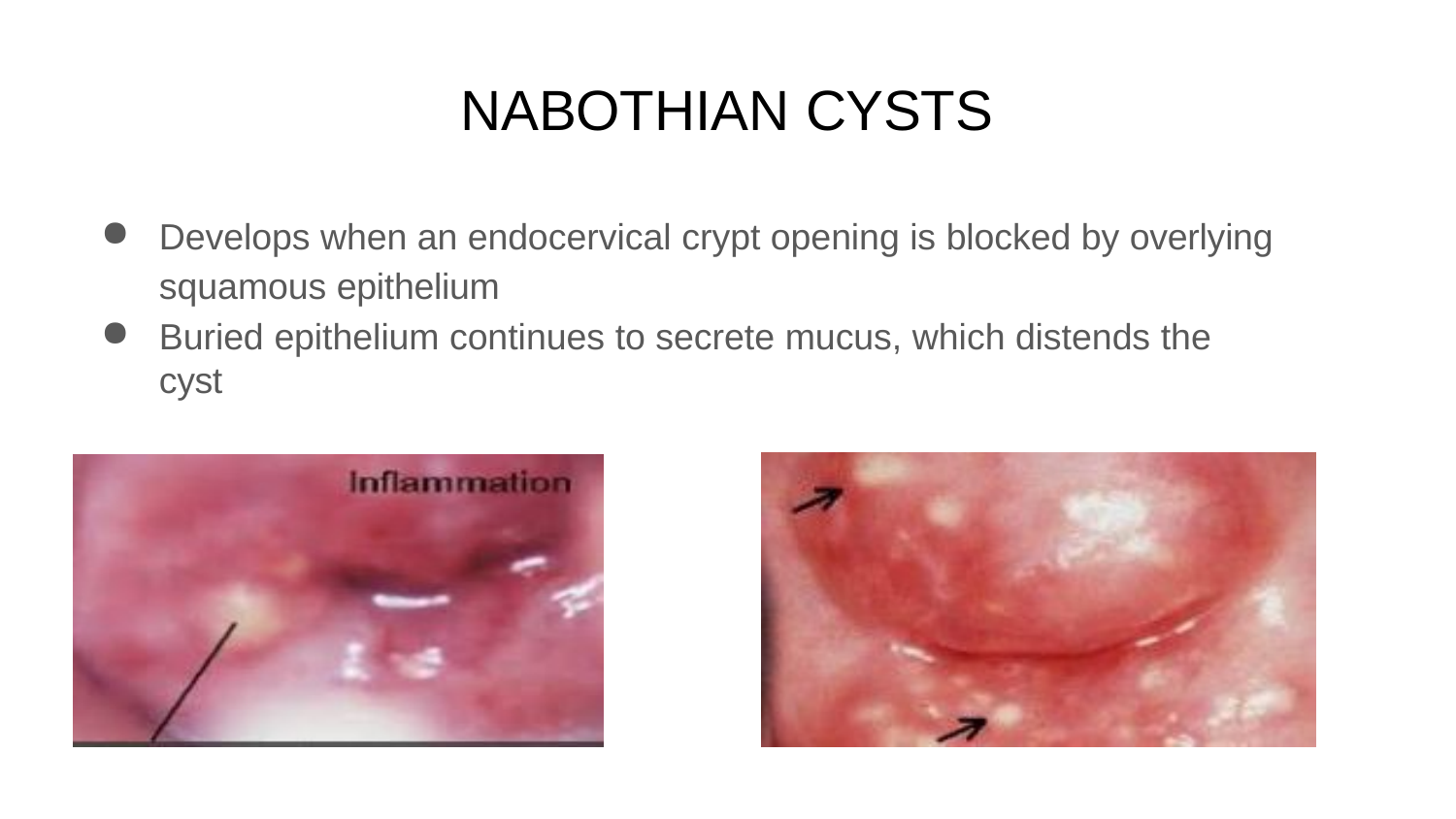

# NABOTHIAN CYSTS
Develops when an endocervical crypt opening is blocked by overlying squamous epithelium
Buried epithelium continues to secrete mucus, which distends the cyst

## Slide 18
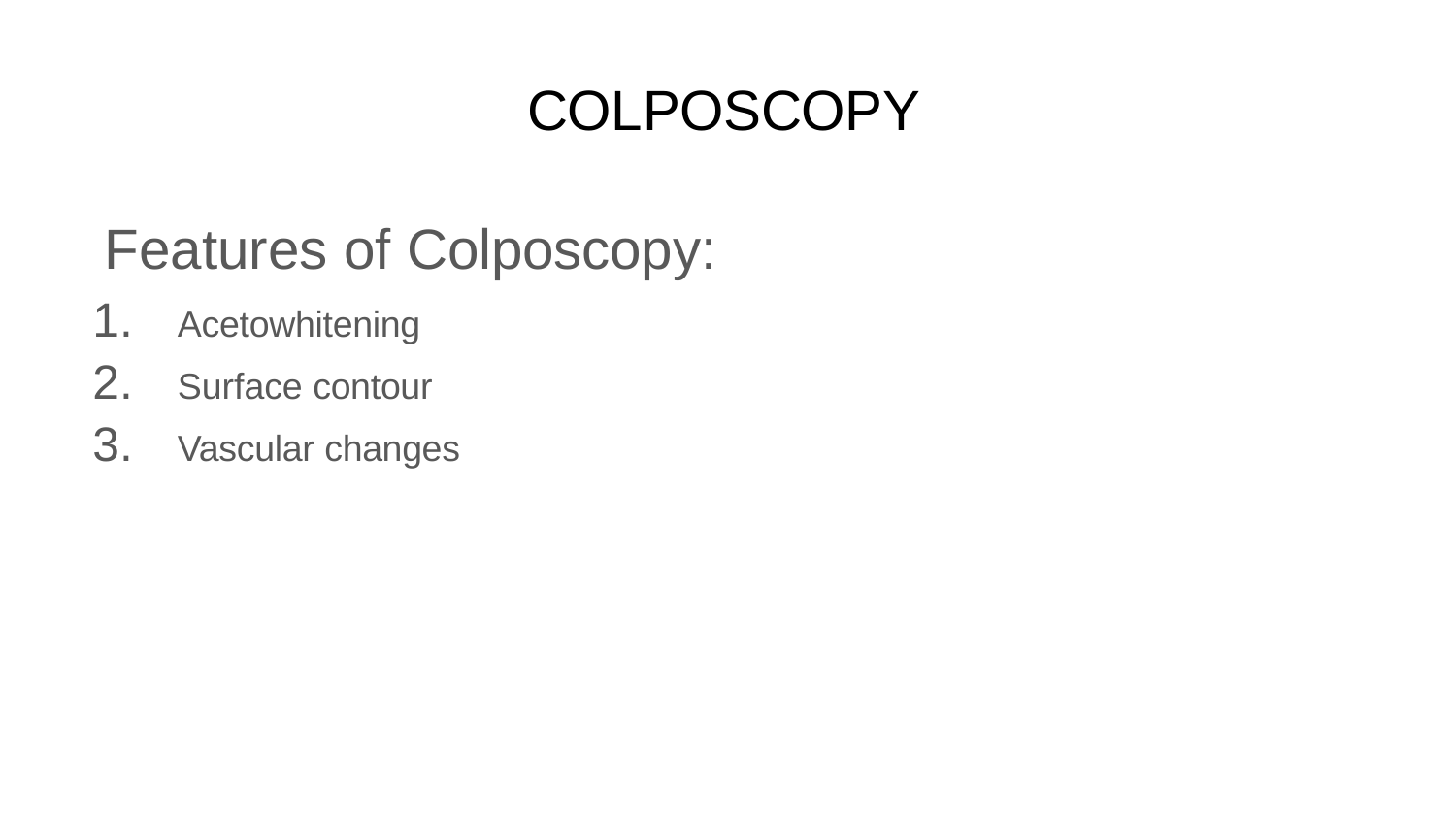

# COLPOSCOPY
Features of Colposcopy:
Acetowhitening
Surface contour
Vascular changes

## Slide 19
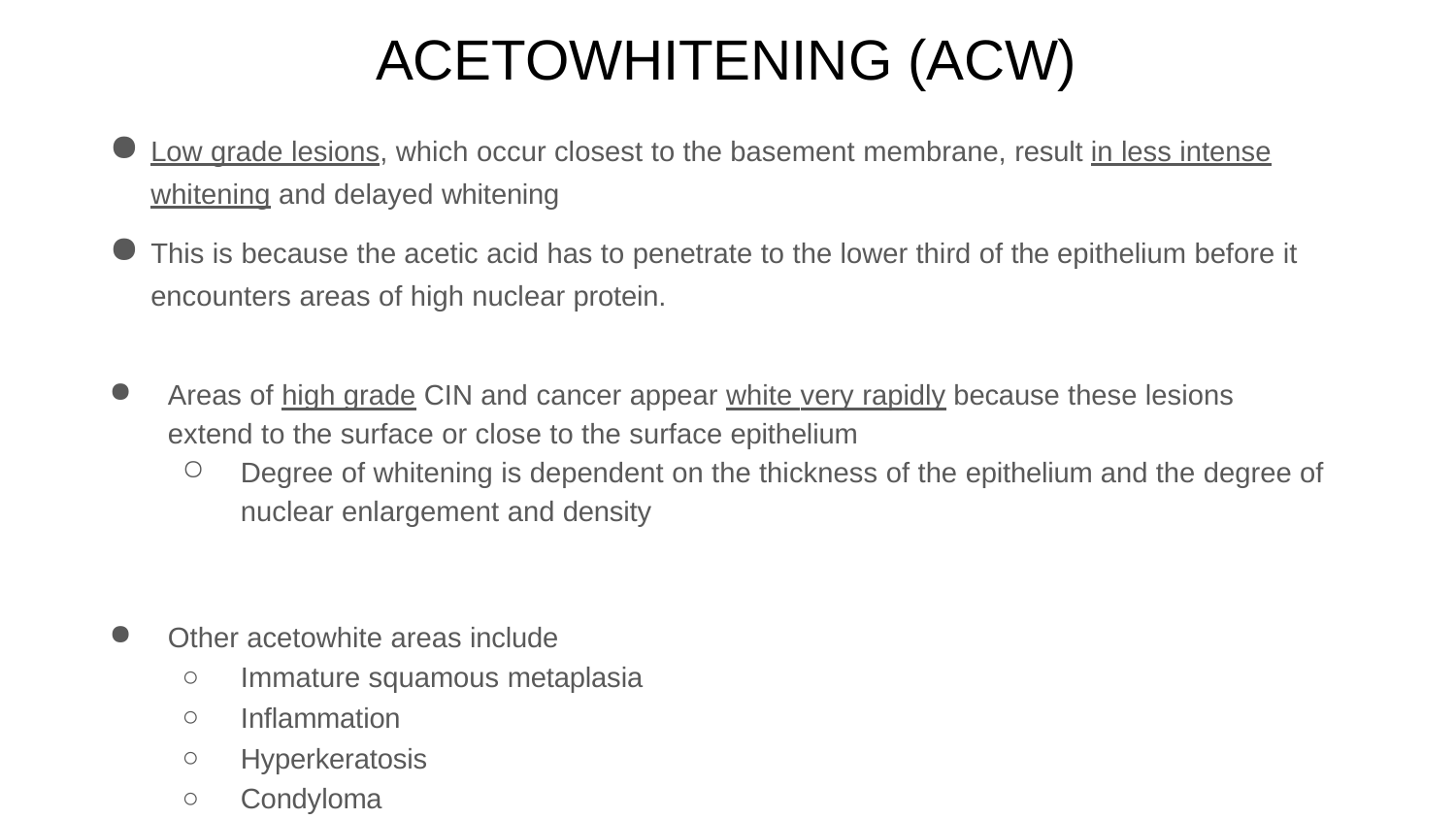

# ACETOWHITENING (ACW)
Low grade lesions, which occur closest to the basement membrane, result in less intense whitening and delayed whitening
This is because the acetic acid has to penetrate to the lower third of the epithelium before it encounters areas of high nuclear protein.
Areas of high grade CIN and cancer appear white very rapidly because these lesions extend to the surface or close to the surface epithelium
Degree of whitening is dependent on the thickness of the epithelium and the degree of nuclear enlargement and density
Other acetowhite areas include
Immature squamous metaplasia
Inflammation
Hyperkeratosis
Condyloma

## Slide 20
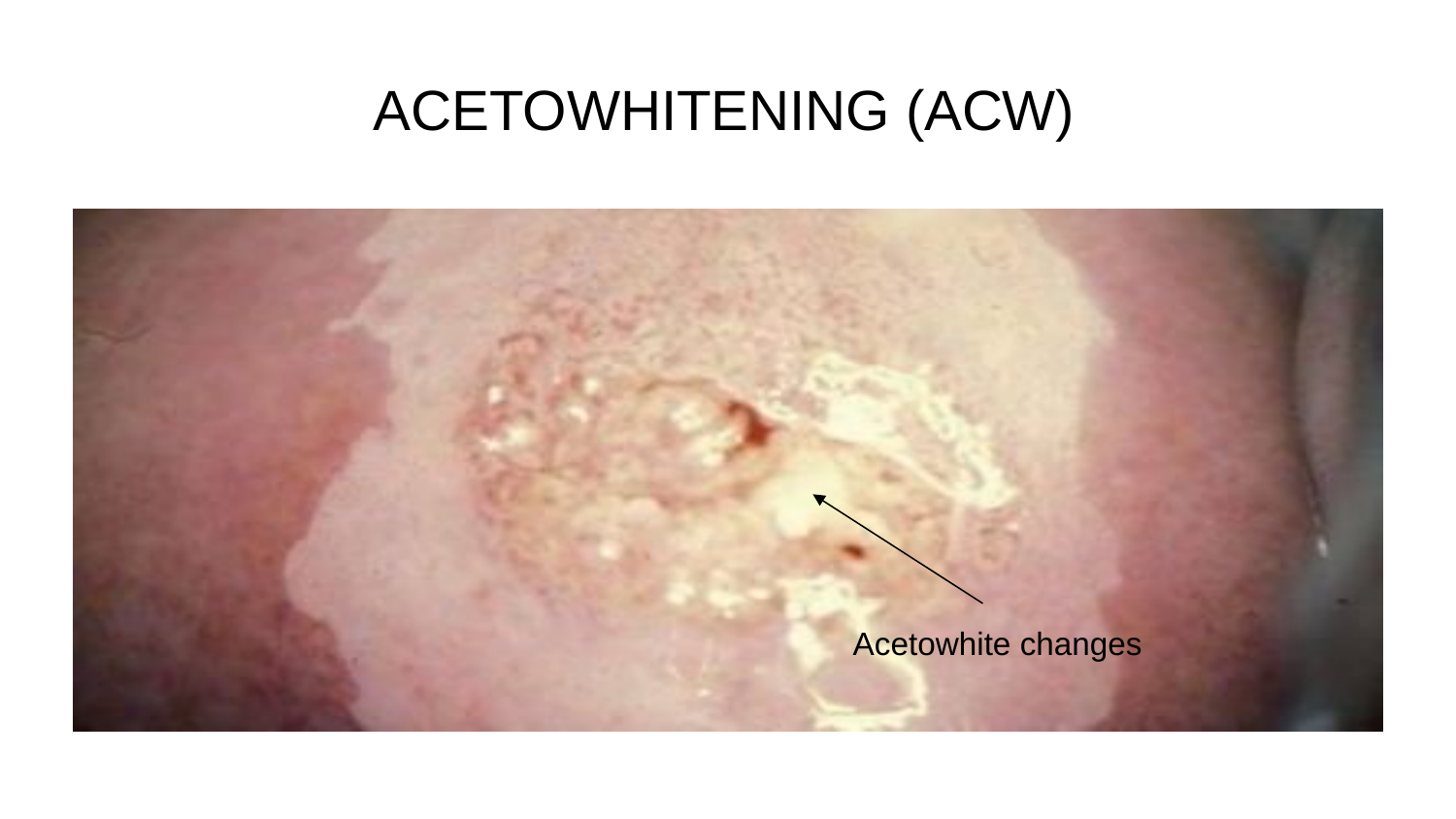

# ACETOWHITENING (ACW)
Acetowhite changes

## Slide 21
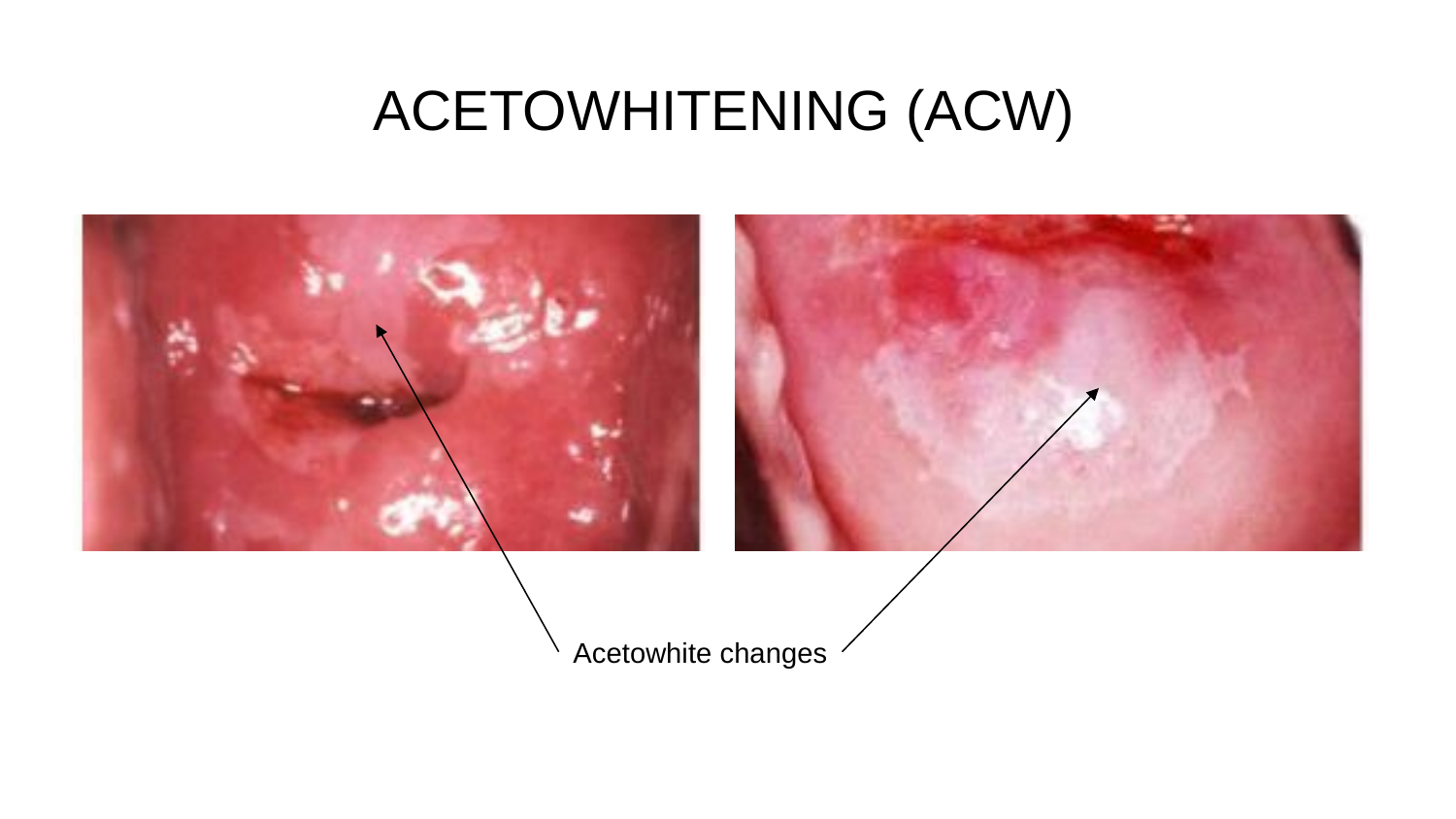

# ACETOWHITENING (ACW)
Acetowhite changes

## Slide 22
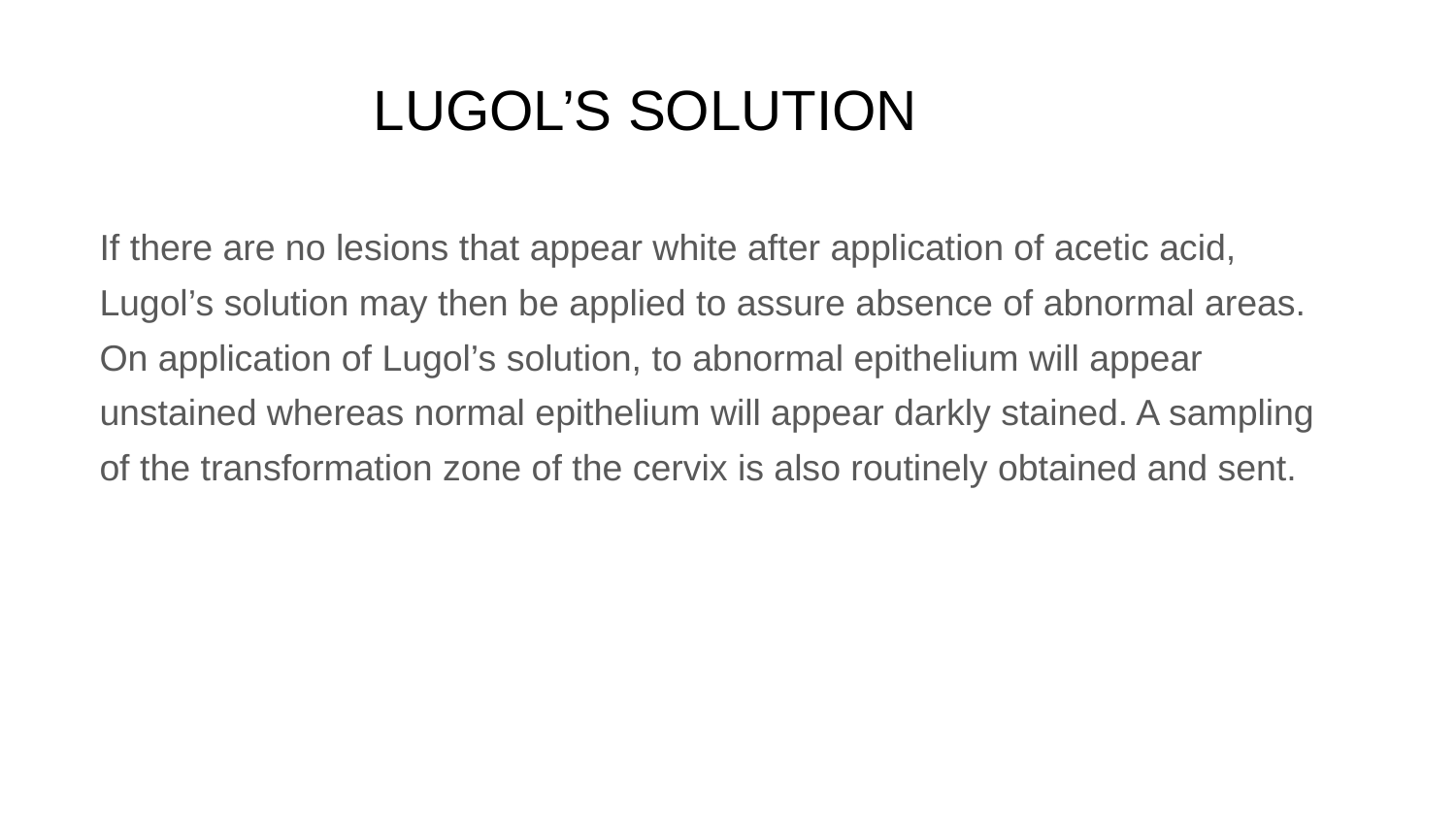

# LUGOL’S SOLUTION
If there are no lesions that appear white after application of acetic acid, Lugol’s solution may then be applied to assure absence of abnormal areas. On application of Lugol’s solution, to abnormal epithelium will appear unstained whereas normal epithelium will appear darkly stained. A sampling of the transformation zone of the cervix is also routinely obtained and sent.

## Slide 23
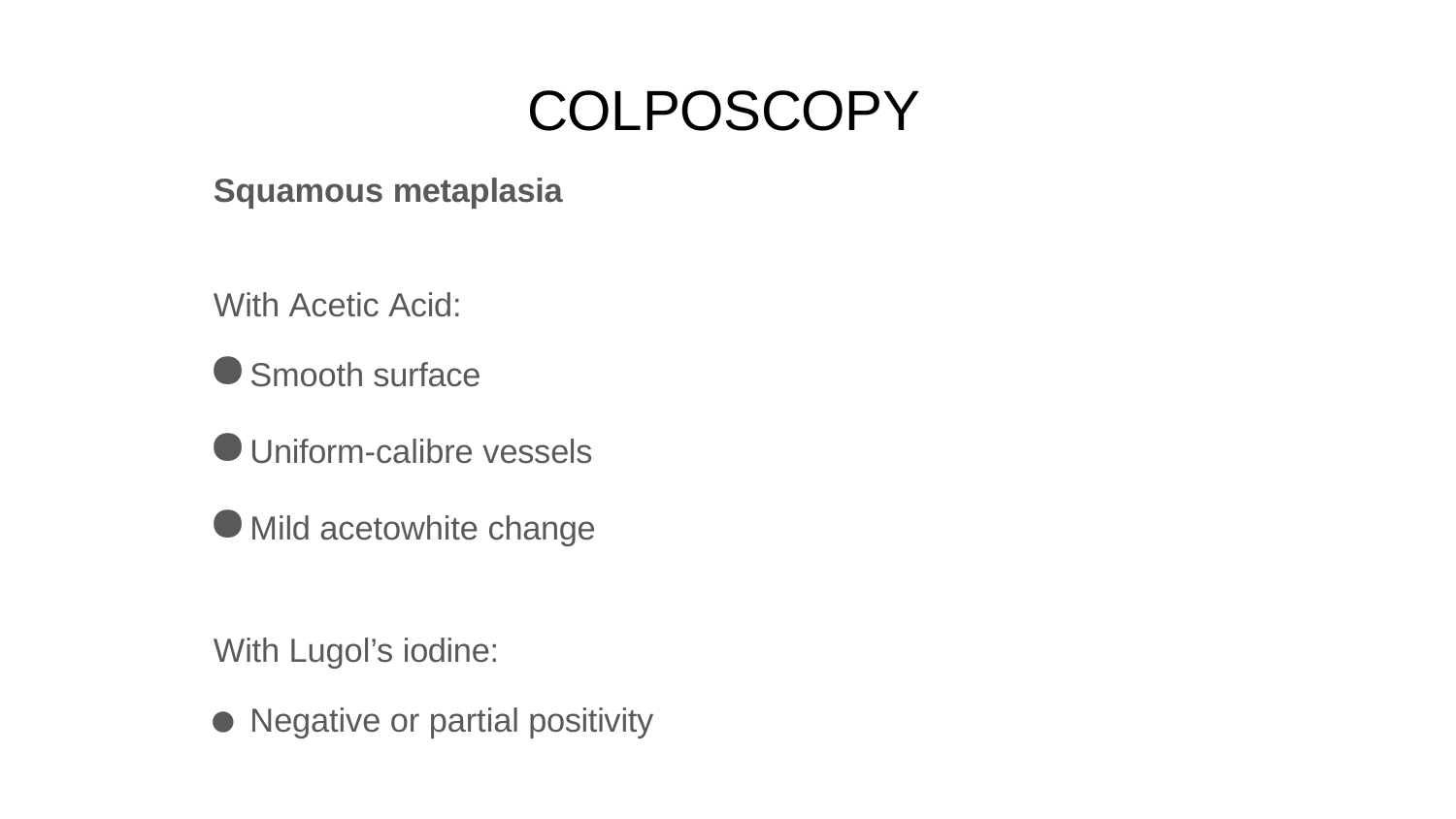

# COLPOSCOPY
Squamous metaplasia
With Acetic Acid:
Smooth surface
Uniform-calibre vessels
Mild acetowhite change
With Lugol’s iodine:
Negative or partial positivity

## Slide 24
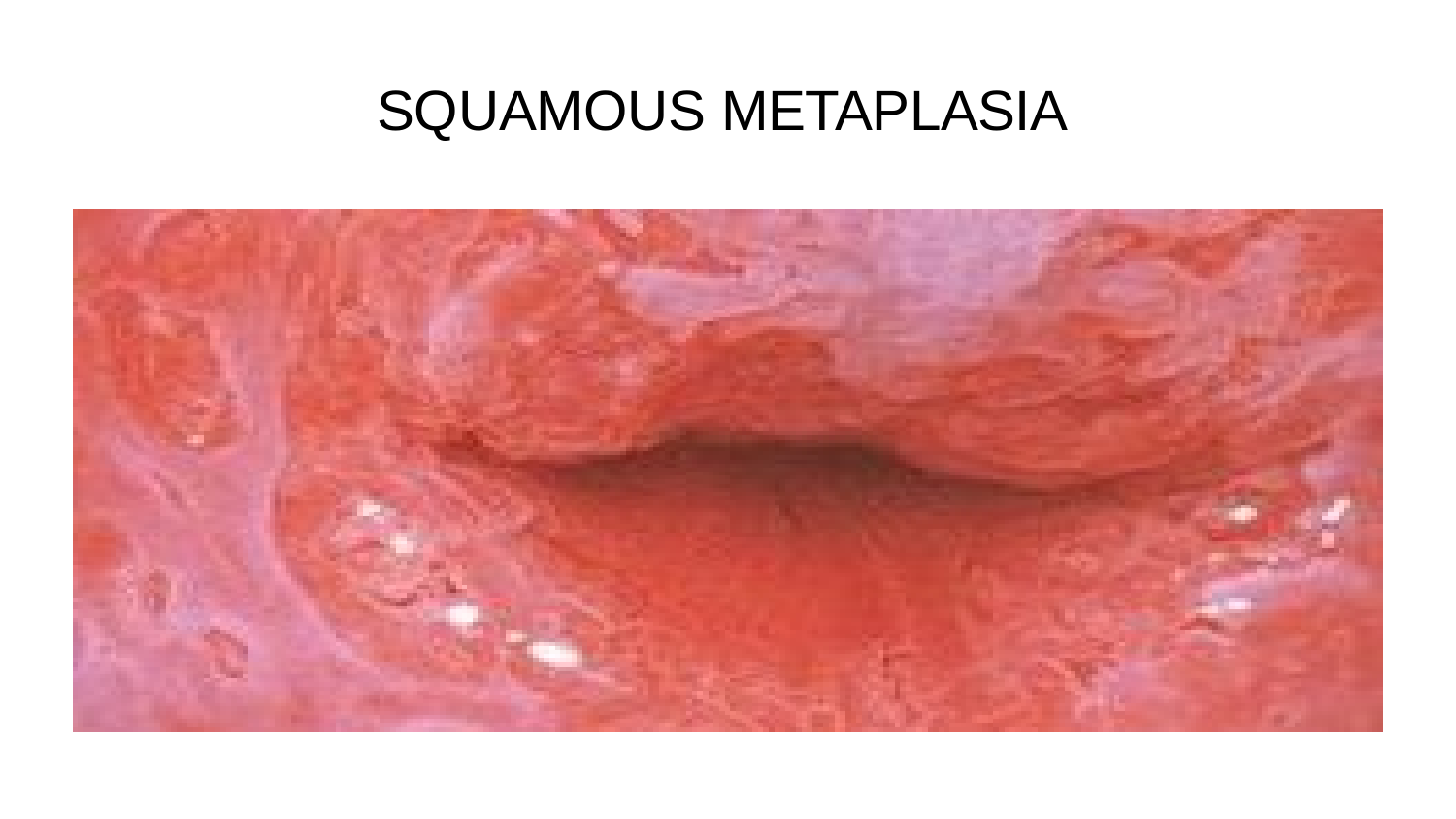

# SQUAMOUS METAPLASIA

## Slide 25
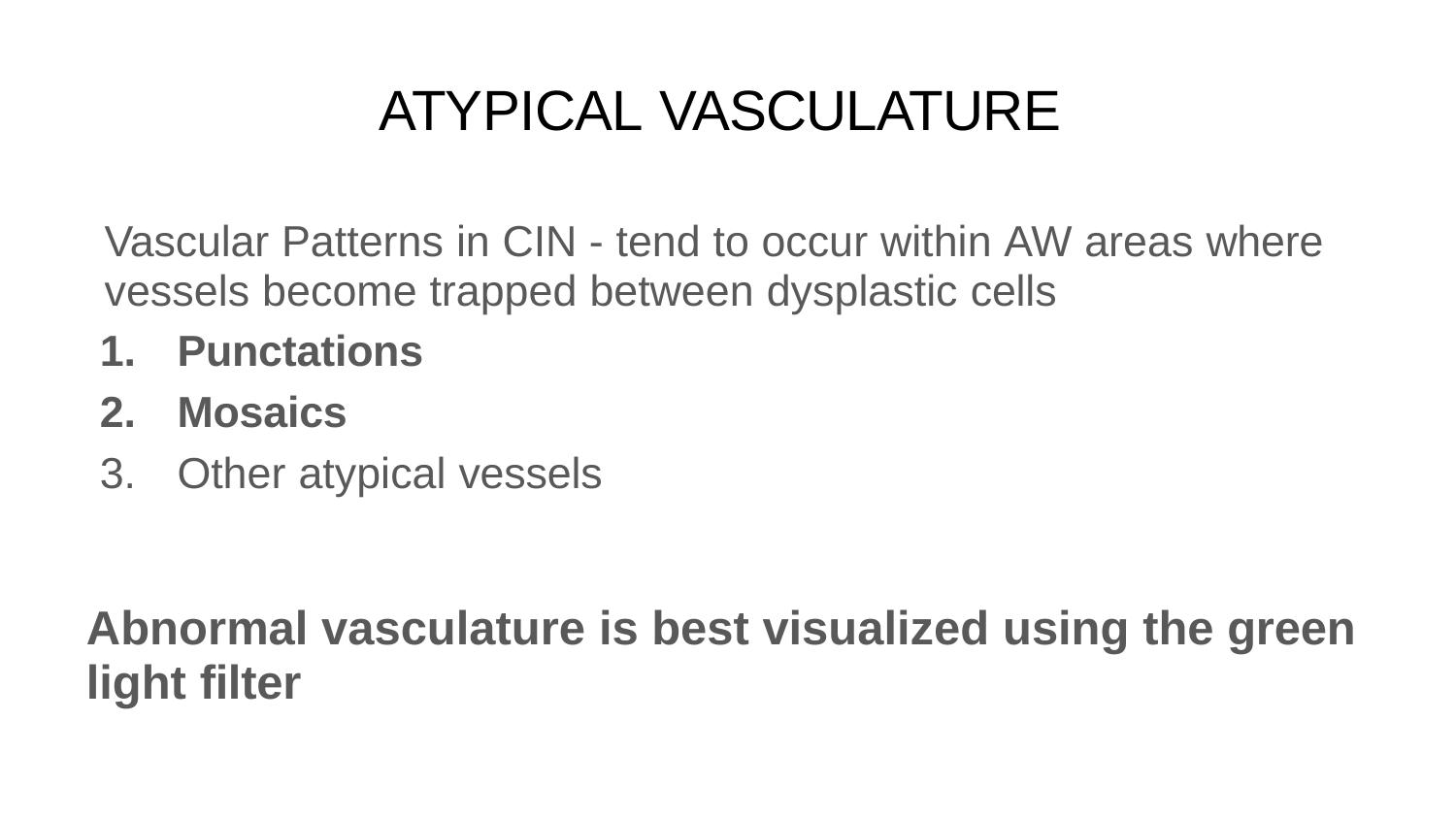

# ATYPICAL VASCULATURE
Vascular Patterns in CIN - tend to occur within AW areas where vessels become trapped between dysplastic cells
Punctations
Mosaics
Other atypical vessels
Abnormal vasculature is best visualized using the green light filter

## Slide 26
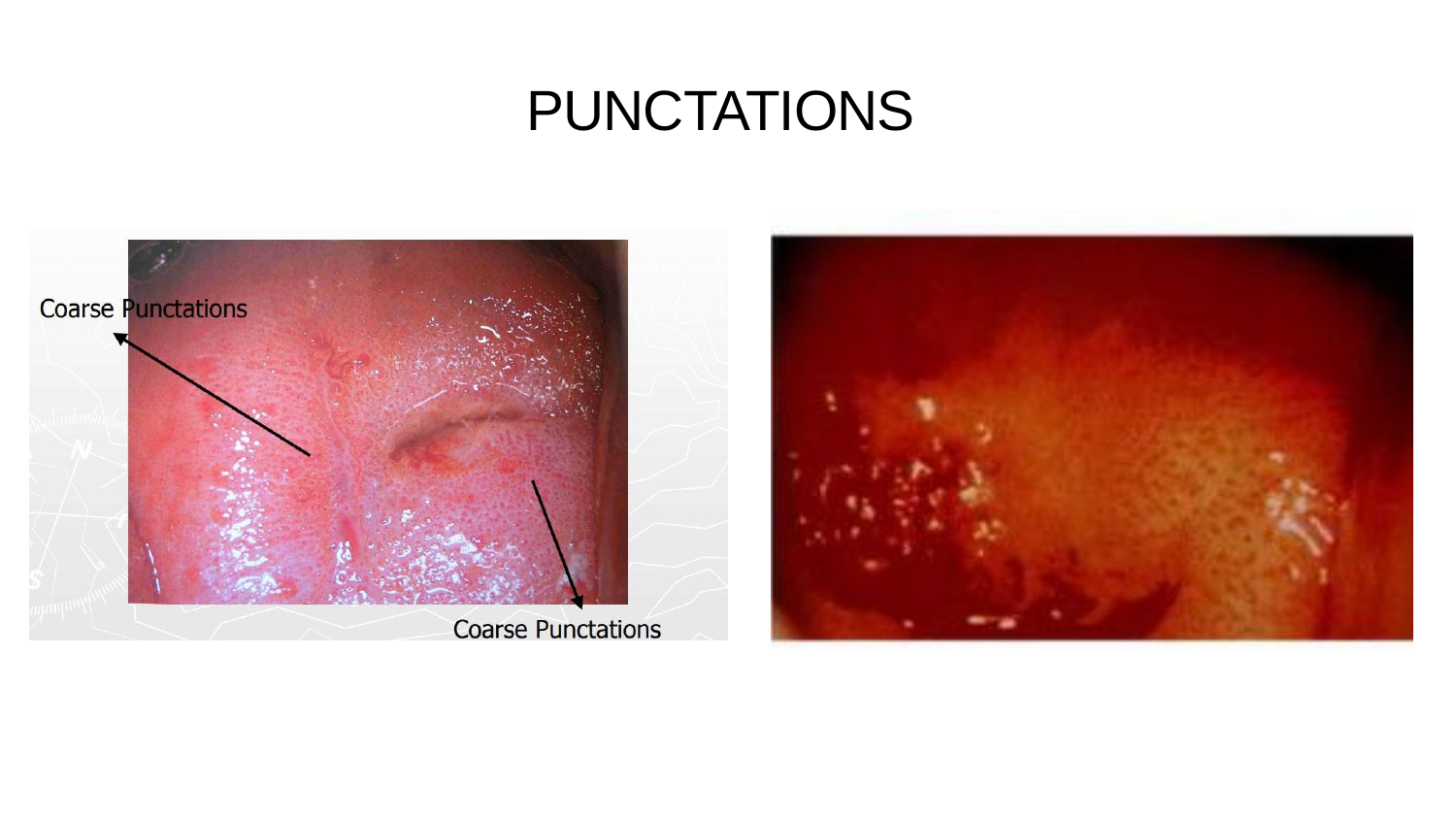

# PUNCTATIONS

## Slide 27
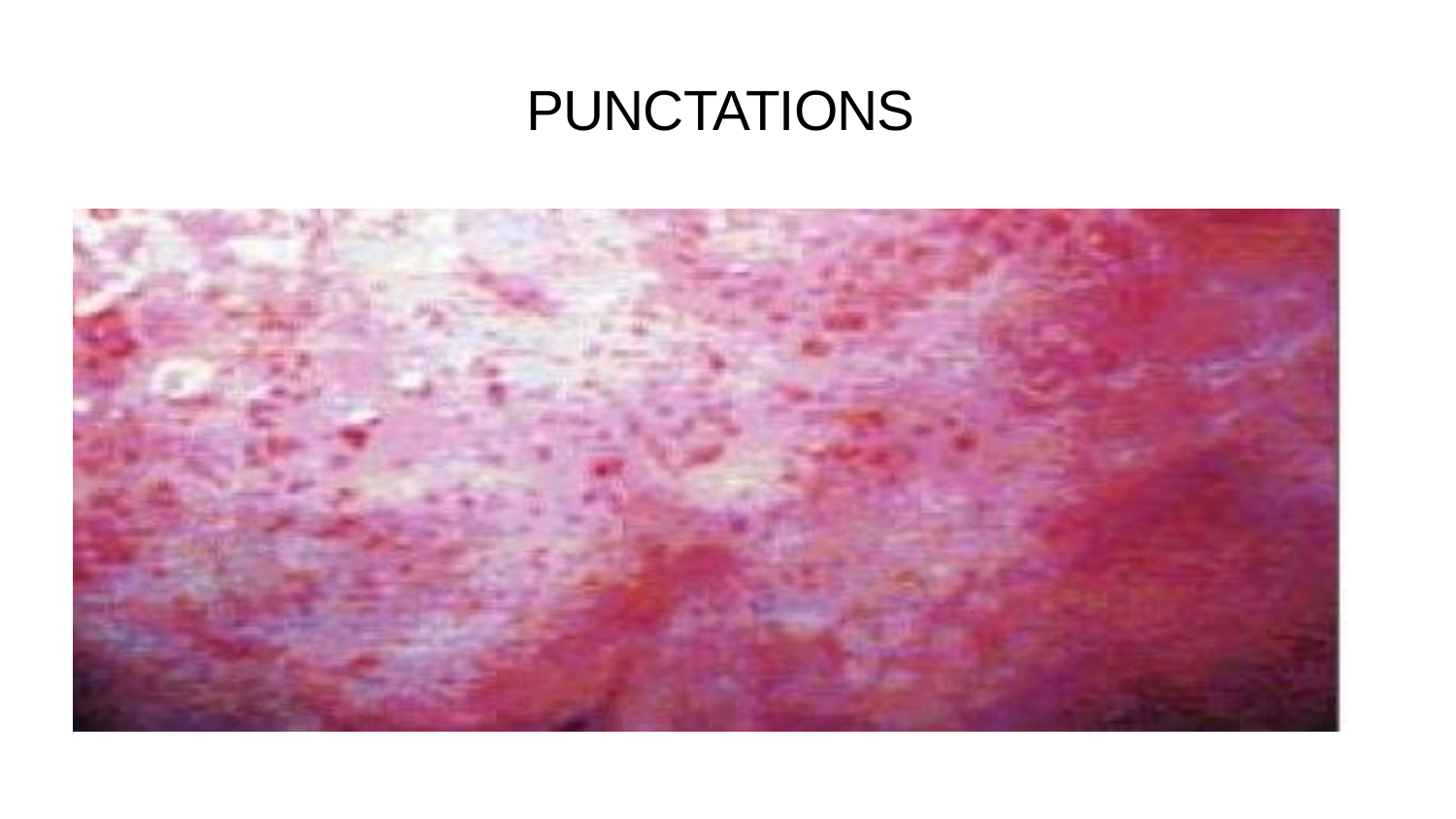

# PUNCTATIONS

## Slide 28
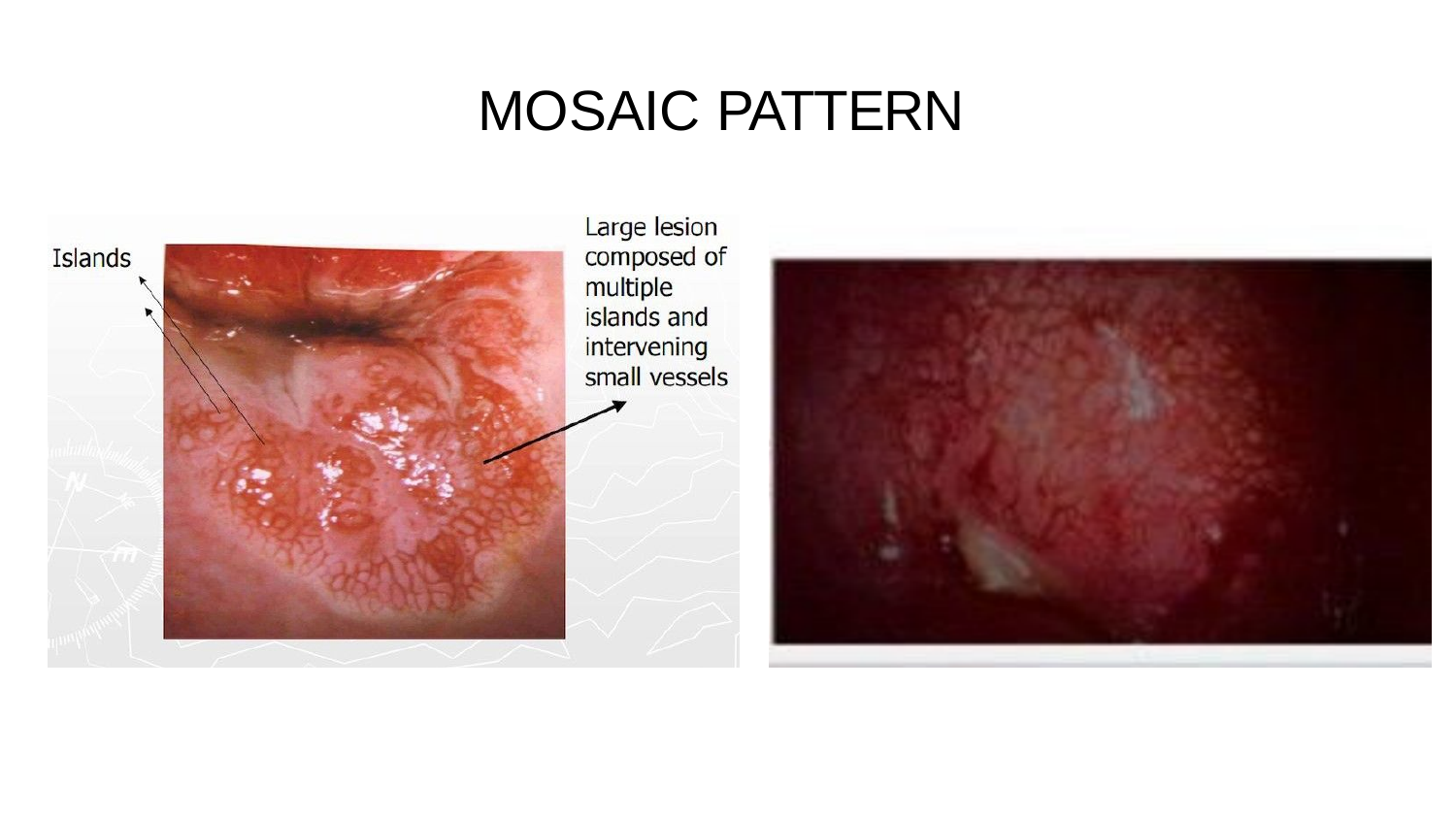

# MOSAIC PATTERN

## Slide 29
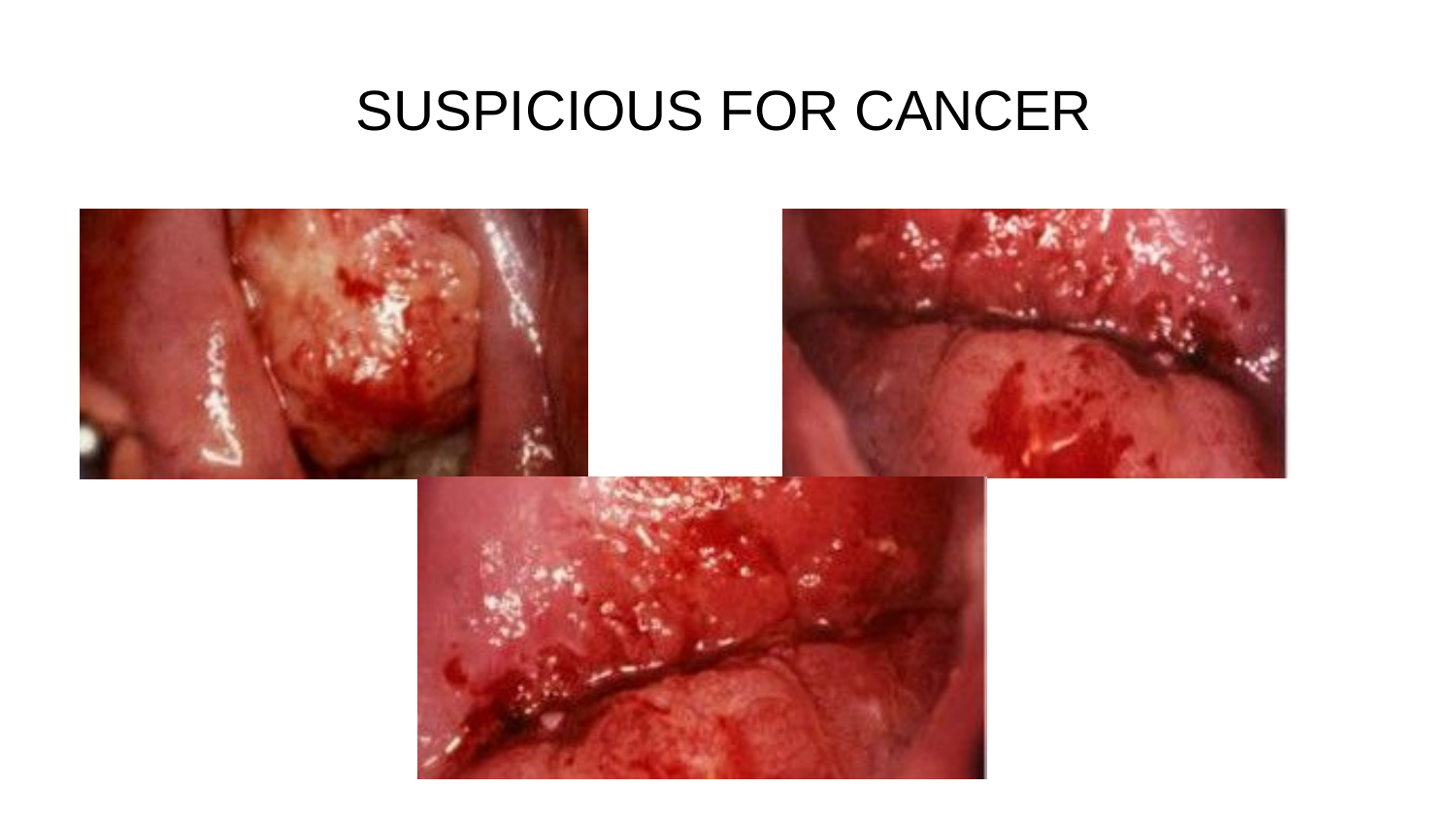

# SUSPICIOUS FOR CANCER

## Slide 30
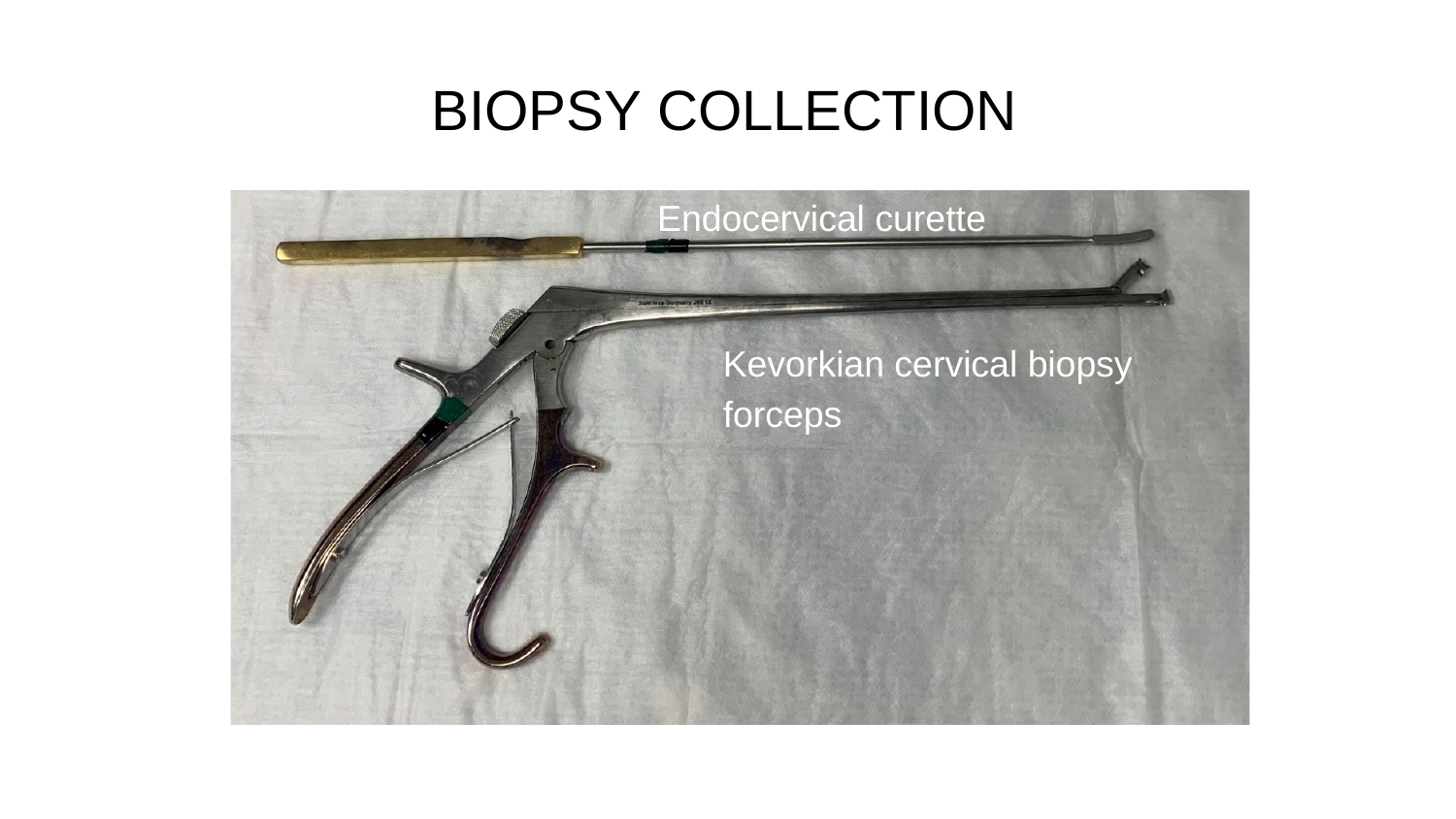

# BIOPSY COLLECTION
Endocervical curette
Kevorkian cervical biopsy forceps

## Slide 31
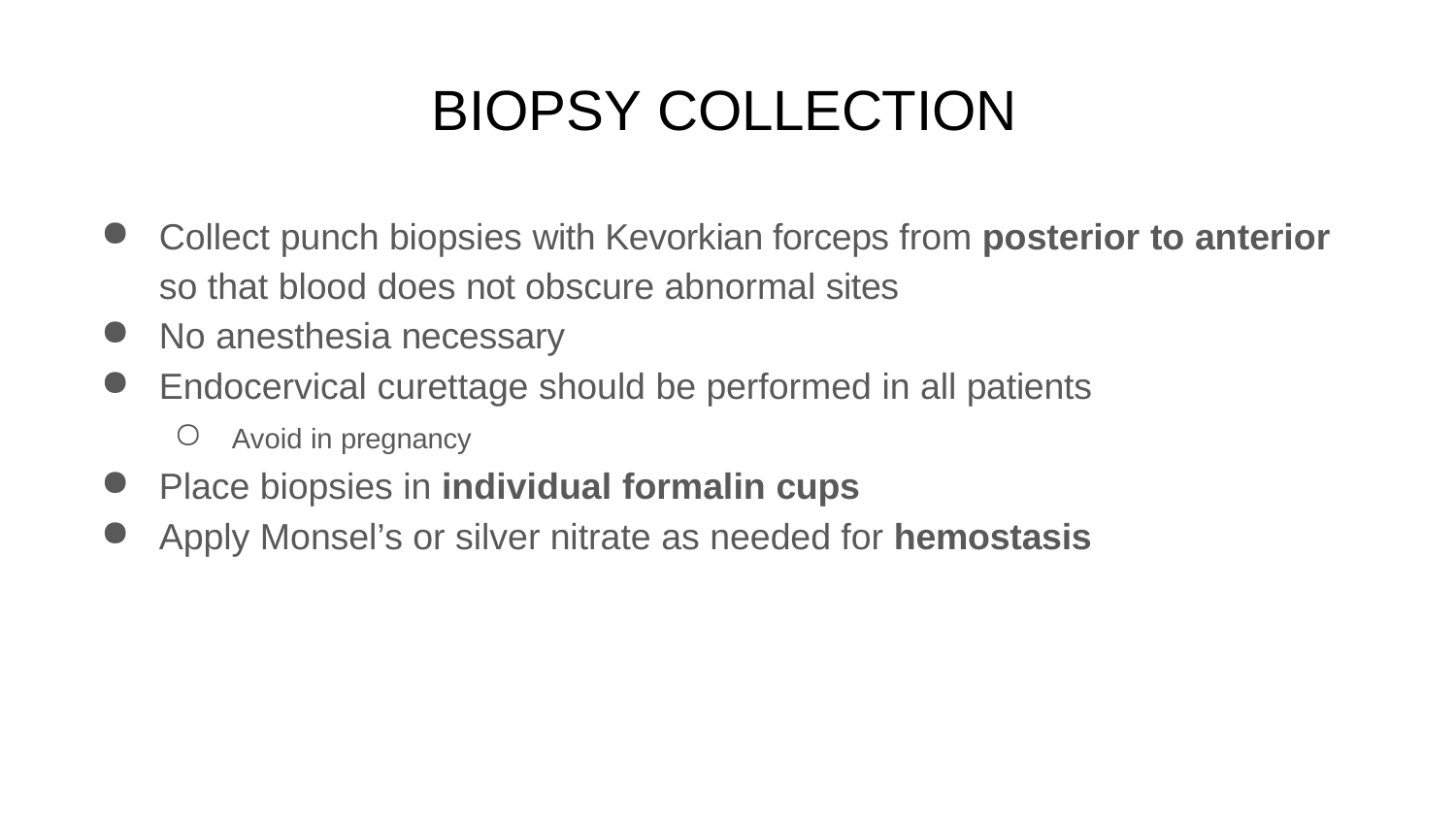

# BIOPSY COLLECTION
Collect punch biopsies with Kevorkian forceps from posterior to anterior so that blood does not obscure abnormal sites
No anesthesia necessary
Endocervical curettage should be performed in all patients
Avoid in pregnancy
Place biopsies in individual formalin cups
Apply Monsel’s or silver nitrate as needed for hemostasis

## Slide 32
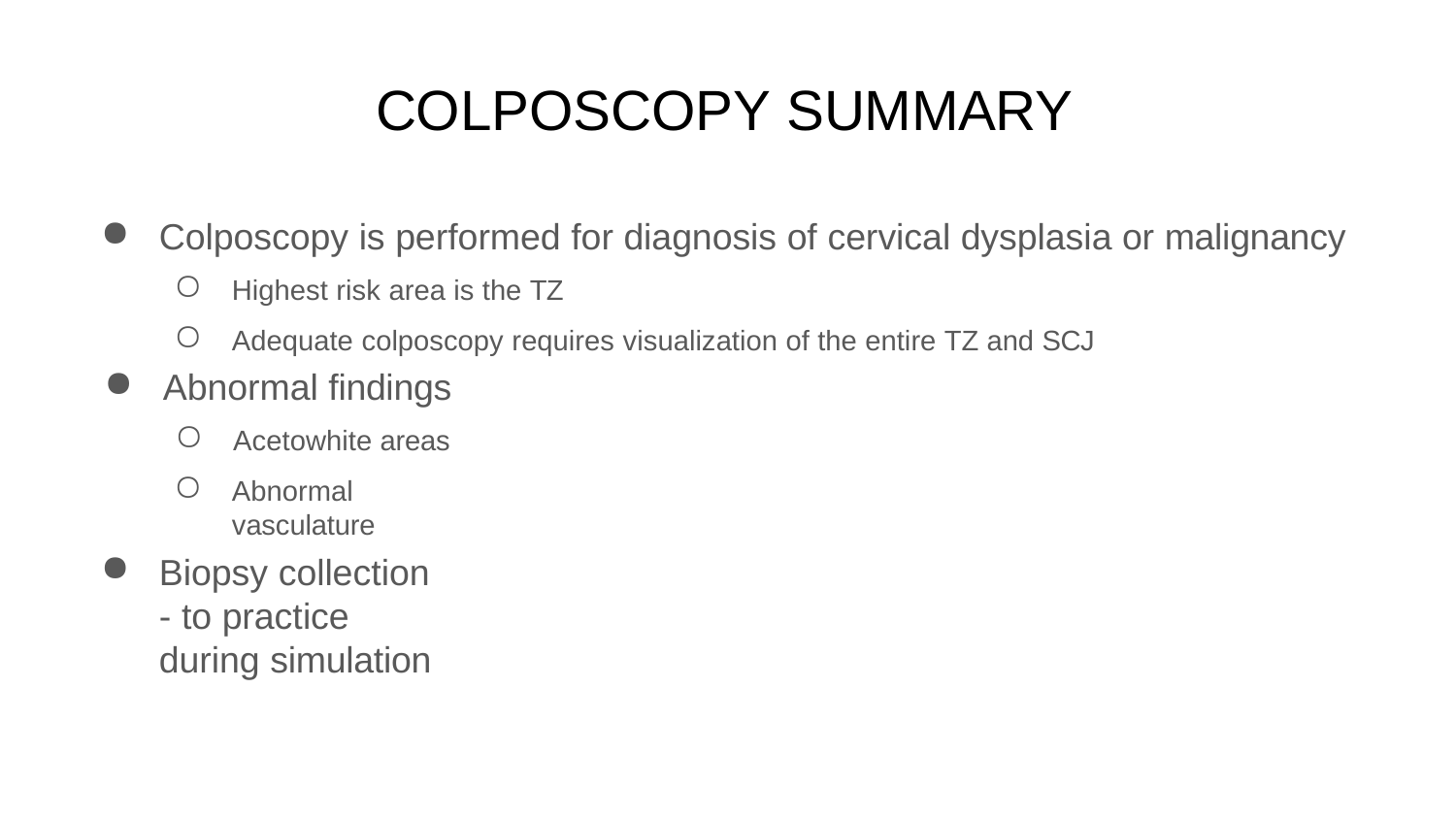

# COLPOSCOPY SUMMARY
Colposcopy is performed for diagnosis of cervical dysplasia or malignancy
Highest risk area is the TZ
Adequate colposcopy requires visualization of the entire TZ and SCJ
Abnormal findings
Acetowhite areas
Abnormal vasculature
Biopsy collection - to practice during simulation

## Slide 33
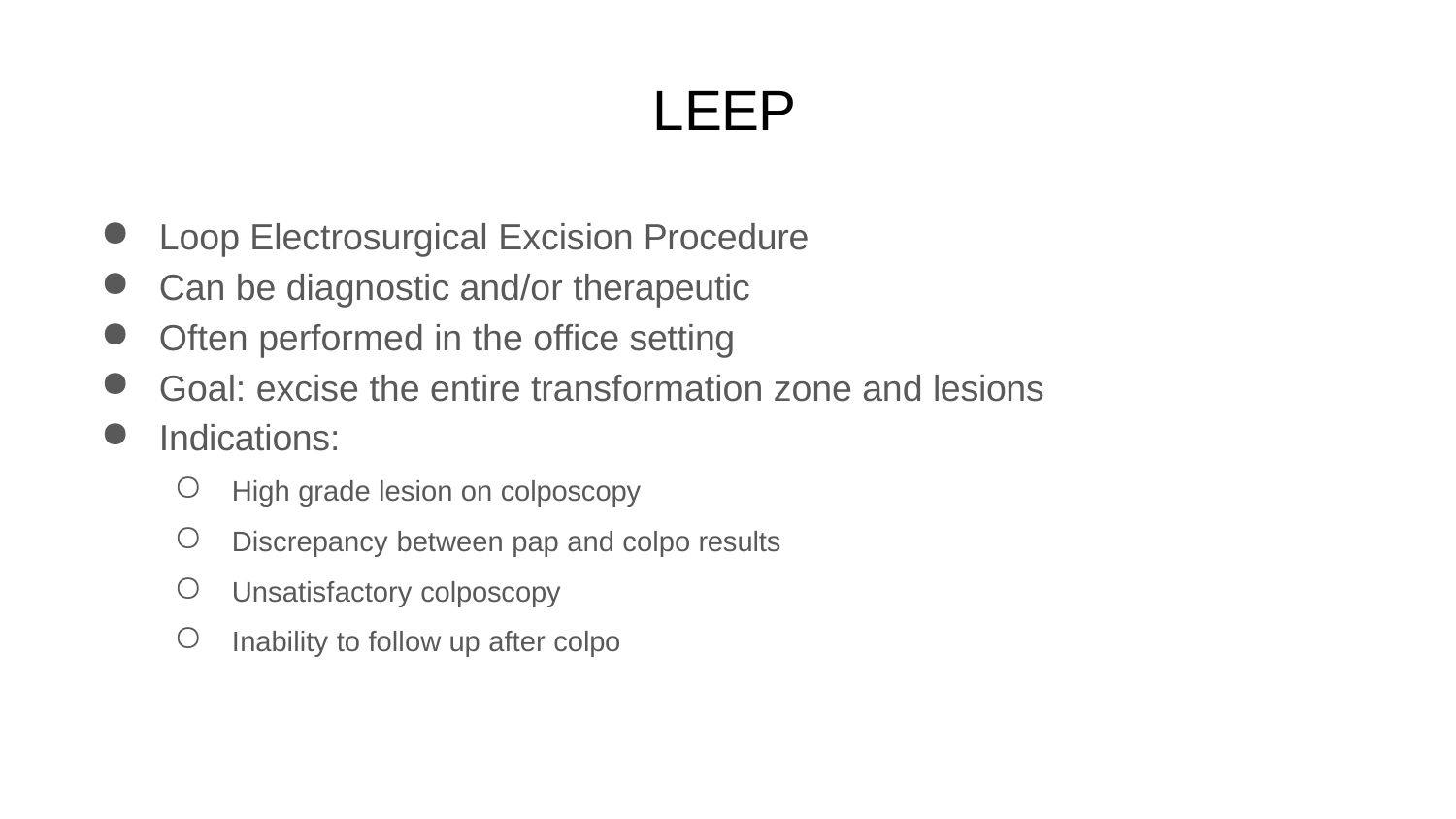

# LEEP
Loop Electrosurgical Excision Procedure
Can be diagnostic and/or therapeutic
Often performed in the office setting
Goal: excise the entire transformation zone and lesions
Indications:
High grade lesion on colposcopy
Discrepancy between pap and colpo results
Unsatisfactory colposcopy
Inability to follow up after colpo

## Slide 34
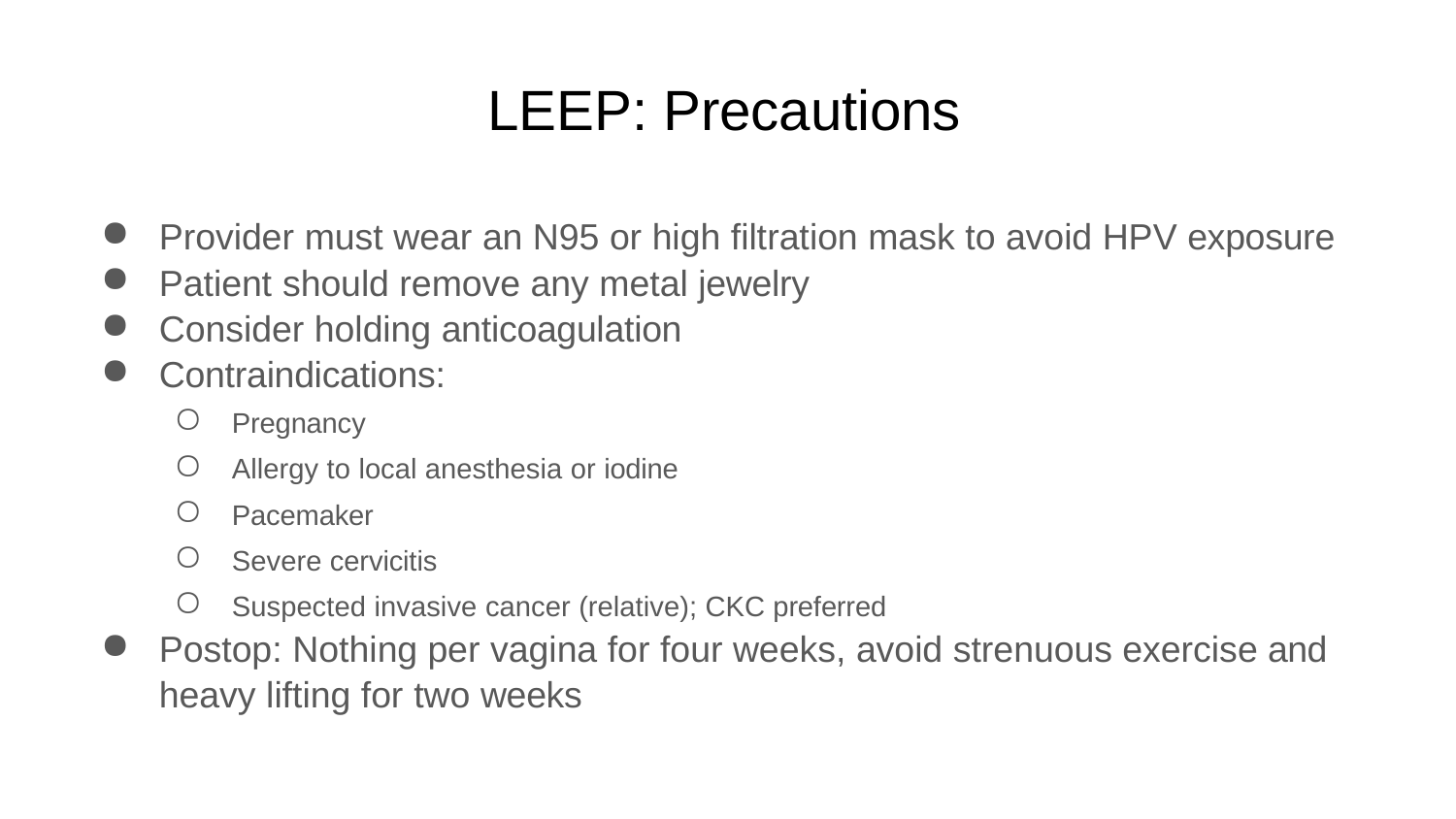

# LEEP: Precautions
Provider must wear an N95 or high filtration mask to avoid HPV exposure
Patient should remove any metal jewelry
Consider holding anticoagulation
Contraindications:
Pregnancy
Allergy to local anesthesia or iodine
Pacemaker
Severe cervicitis
Suspected invasive cancer (relative); CKC preferred
Postop: Nothing per vagina for four weeks, avoid strenuous exercise and heavy lifting for two weeks

## Slide 35
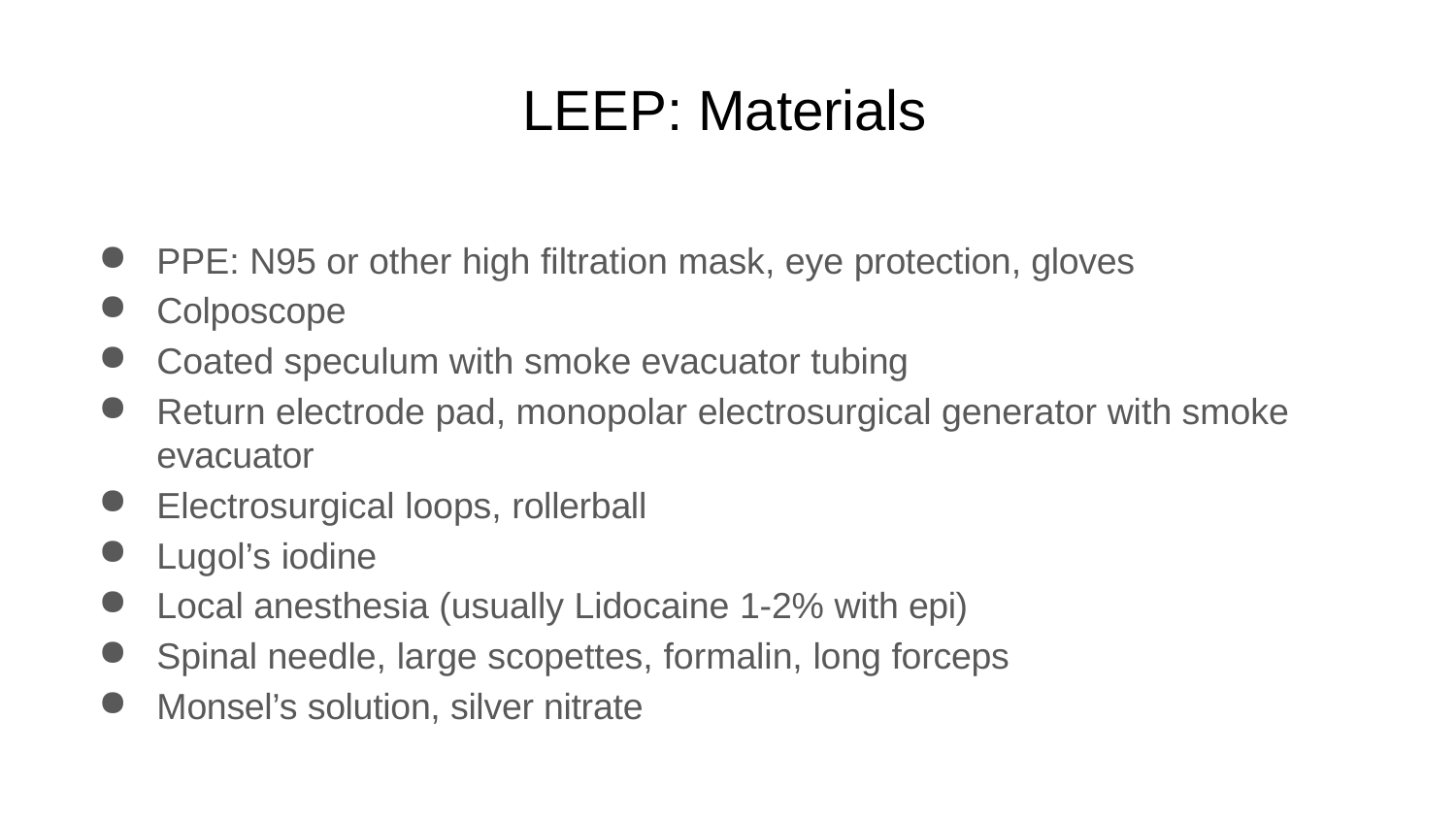

# LEEP: Materials
PPE: N95 or other high filtration mask, eye protection, gloves
Colposcope
Coated speculum with smoke evacuator tubing
Return electrode pad, monopolar electrosurgical generator with smoke evacuator
Electrosurgical loops, rollerball
Lugol’s iodine
Local anesthesia (usually Lidocaine 1-2% with epi)
Spinal needle, large scopettes, formalin, long forceps
Monsel’s solution, silver nitrate

## Slide 36
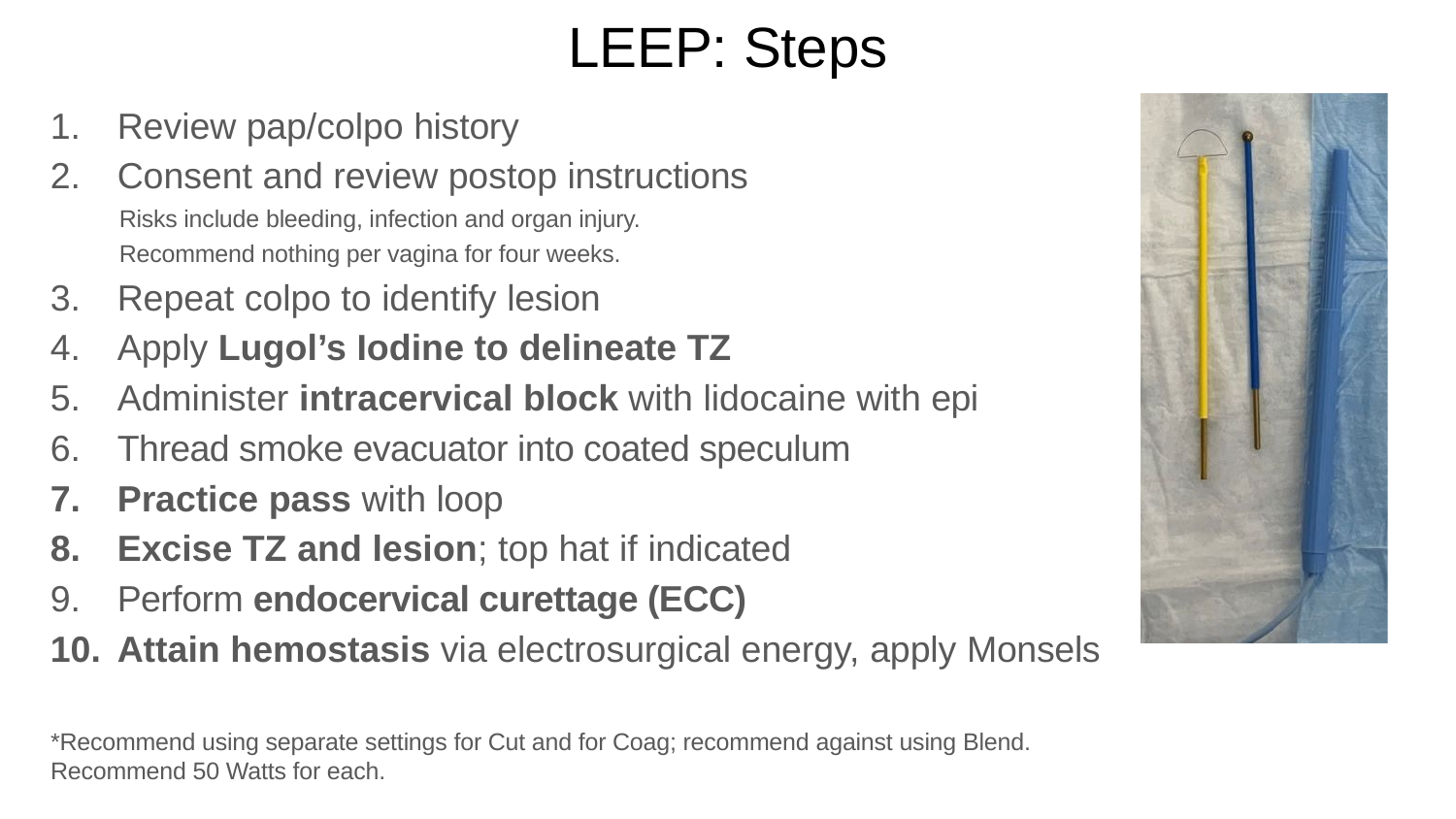

# LEEP: Steps
Review pap/colpo history
Consent and review postop instructions
	Risks include bleeding, infection and organ injury.
	Recommend nothing per vagina for four weeks.
Repeat colpo to identify lesion
Apply Lugol’s Iodine to delineate TZ
Administer intracervical block with lidocaine with epi
Thread smoke evacuator into coated speculum
Practice pass with loop
Excise TZ and lesion; top hat if indicated
Perform endocervical curettage (ECC)
Attain hemostasis via electrosurgical energy, apply Monsels
*Recommend using separate settings for Cut and for Coag; recommend against using Blend. Recommend 50 Watts for each.

## Slide 37
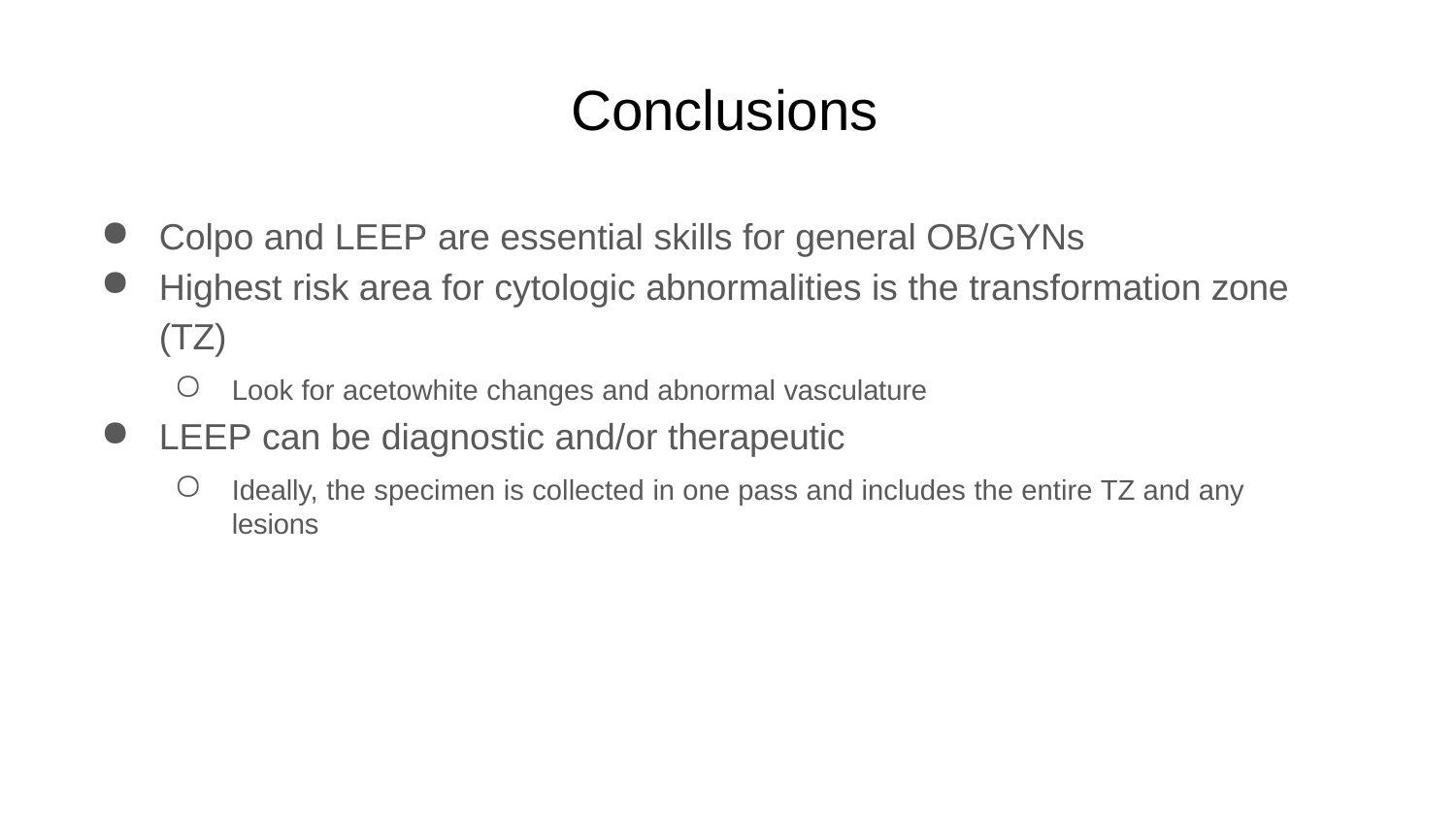

# Conclusions
Colpo and LEEP are essential skills for general OB/GYNs
Highest risk area for cytologic abnormalities is the transformation zone (TZ)
Look for acetowhite changes and abnormal vasculature
LEEP can be diagnostic and/or therapeutic
Ideally, the specimen is collected in one pass and includes the entire TZ and any lesions

## Slide 38
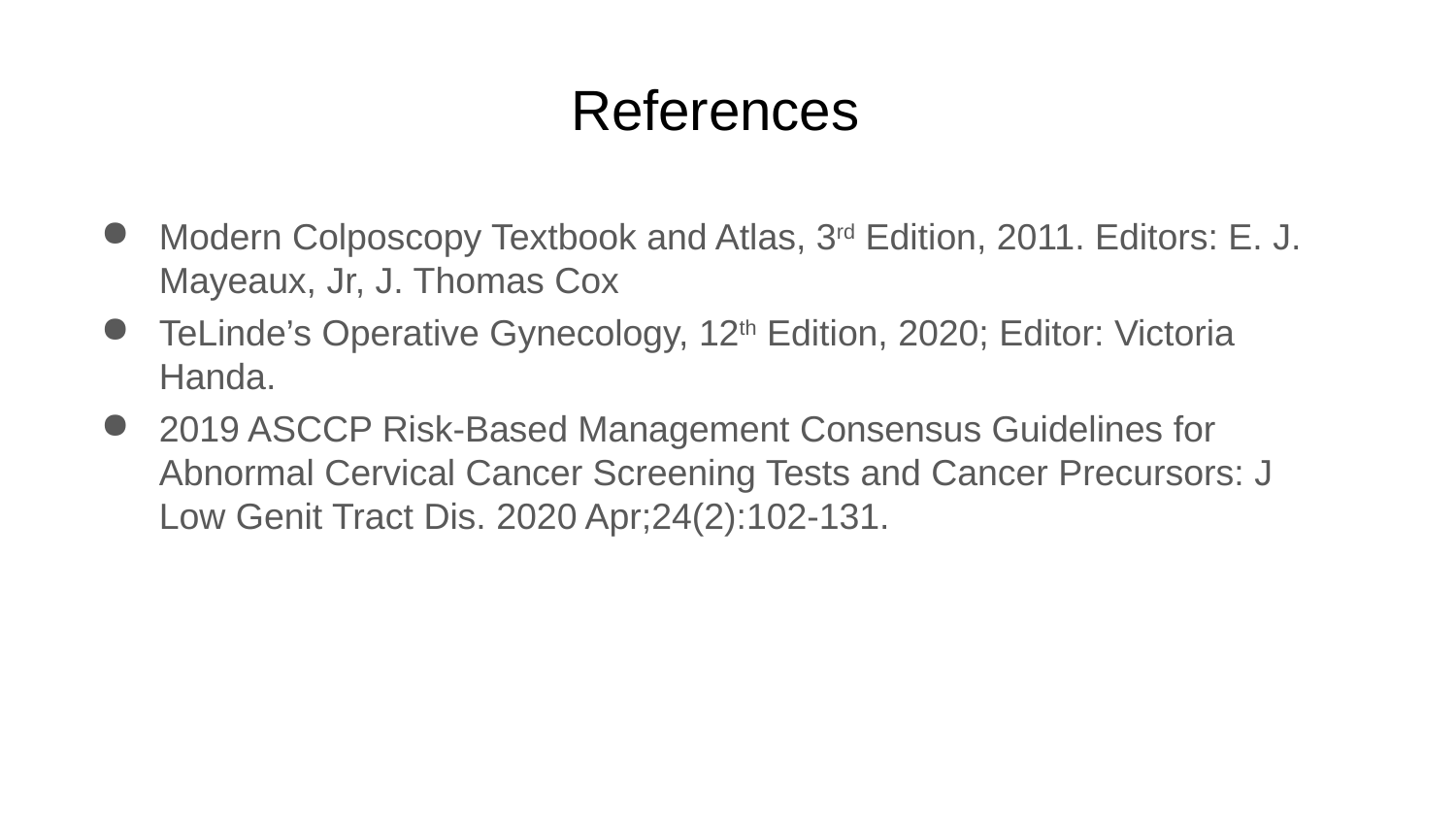

# References
Modern Colposcopy Textbook and Atlas, 3rd Edition, 2011. Editors: E. J. Mayeaux, Jr, J. Thomas Cox
TeLinde’s Operative Gynecology, 12th Edition, 2020; Editor: Victoria Handa.
2019 ASCCP Risk-Based Management Consensus Guidelines for Abnormal Cervical Cancer Screening Tests and Cancer Precursors: J Low Genit Tract Dis. 2020 Apr;24(2):102-131.
